# Past continental shelf evolution increased Antarctic ice sheet sensitivity to climatic conditions

**DOI:** 10.1038/s41598-018-29718-7

**Published:** 2018-07-27

**Authors:** Florence Colleoni, Laura De Santis, Enea Montoli, Elisabetta Olivo, Christopher C. Sorlien, Philip J. Bart, Edward G. W. Gasson, Andrea Bergamasco, Chiara Sauli, Nigel Wardell, Stefano Prato

**Affiliations:** 1 Fondazione Centro Euro-Mediterraneo sui Cambiamenti Climatici, Bologna, 40128 Italy; 2 Istituto Nazionale di Oceanografia Sperimentale, Sgonico, 34010 Italy; 3 Dipartimento Scienze Fisiche, della Terra e dell’Ambiente, Universita di Siena, 53100 Siena, Italy; 4 Earth Research Institute, University of California, Santa Barbara, California 93106 USA; 5 Louisiana State University, Department of Geology and Geophysics, Baton Rouge, Louisiana 70803 USA; 6 Department of Geography, University of Sheffield, Sheffield, S10 2TN UK; 7 CNR-ISMAR, Arsenale Tesa 104, Castello 2737/F, Venice, 30122 Italy; 8 A.P.E. Research srl, Area Science Park, Basovizza Campus, Trieste, 34149 Italy

**Keywords:** Palaeoclimate, Computational science

## Abstract

Over the past 34 Million years, the Antarctic continental shelf has gradually deepened due to ice sheet loading, thermal subsidence, and erosion from repeated glaciations. The deepening that is recorded in the sedimentary deposits around the Antarctic margin indicates that after the mid-Miocene Climate Optimum (≈15 Ma), Antarctic Ice Sheet (AIS) dynamical response to climate conditions changed. We explore end-members for maximum AIS extent, based on ice-sheet simulations of a late-Pleistocene and a mid-Miocene glaciation. Fundamental dynamical differences emerge as a consequence of atmospheric forcing, eustatic sea level and continental shelf evolution. We show that the AIS contributed to the amplification of its own sensitivity to ocean forcing by gradually expanding and eroding the continental shelf, that probably changed its tipping points through time. The lack of past topographic and bathymetric reconstructions implies that so far, we still have an incomplete understanding of AIS fast response to past warm climate conditions, which is crucial to constrain its future evolution.

## Introduction

On geological timescales, the dynamic changes of the AIS are likely to be the result of the combination of global climate cooling, the expansion and deepening of the continental shelf, and changes in ocean circulation and temperature. Although the extent of the AIS is primarily determined by the orbital configuration of a given time period^1^, its long-term ice volume results from atmospheric CO_2_ concentration, subglacial topography and continental margin evolution^2,3^. The lack of detailed knowledge of the modern subglacial morphology, especially in key sectors of the AIS, induces large uncertainties in the prediction of the Marine Ice Sheet instability of highly sensitive outlet glaciers or ice shelves around Antarctica^4,5^. Reconstructing past seabed morphology around, and subglacial topography beneath, Antarctica is even more challenging^6^. Past ice sheet reconstructions provide useful information on the response of the AIS to warm and cold climate fluctuations of different magnitudes. However, uncertainties in the AIS contribution to past sea level changes or other forcing mechanisms during key periods, such as the middle Miocene, the middle Pliocene or the Last Interglacial, are still large^7^. A previous study^8^ using a reconstruction of the Eocene-Oligocene topography, proposed that a terrestrial West Antarctic Ice Sheet (WAIS), grounded mostly above sea level, was able to form much earlier than previously thought because of the shallower topography. Other numerical reconstructions of the mid -Pliocene (3 Ma) and present-day AIS volumes also account for the uncertainties linked with bedrock reconstruction^4,9^. In particular, recent simulations of the AIS forced with Miocene climate conditions and using an idealized mid-Miocene bathymetry^2^ show that grounded ice volumes and AIS vulnerability to marine instabilities are strongly influenced by the reconstructed subglacial topography. Similarly, simulated transient expansion of the AIS during the last glacial cycle occurs faster on shallower bathymetries that are shallower than today. Those simulations also show that the timing of expansion depends on the interplay between the magnitude of inland accumulation, ocean warming and calving^3^.

Determining when continental margins deepened sufficiently to change the AIS dynamics and how the ice sheet reacted subsequently to atmospheric and oceanic condition changes is important in identifying useful analogues to future climate change. In this study, we use an ice-sheet model to investigate the impact that the gradual deepening of the Antarctic continental margin had on both Miocene and Pleistocene AIS response to changes in ocean forcing. We show that the AIS amplified its own sensitivity to ocean forcing, by gradually eroding the continental margins. Our results also show that eustatic sea level fall and glacial-isostatic adjustment (GIA) modulate the capability of the ice sheet to pin on bathymetric highs and that the importance of pinning points increases with the deepening of the continental margins. Finally, our simulations show a gradual expansion of the cold-based ice area from the middle Miocene to the Pleistocene that causes fundamental differences in the AIS dynamics. We conclude that as a result of the gradual deepening of the continental margins, ocean forcing became a first-order factor in the AIS expansion and retreat after the middle Miocene.

## Antarctic Paleo-Topographies and Bathymetries

**Figure 1:**
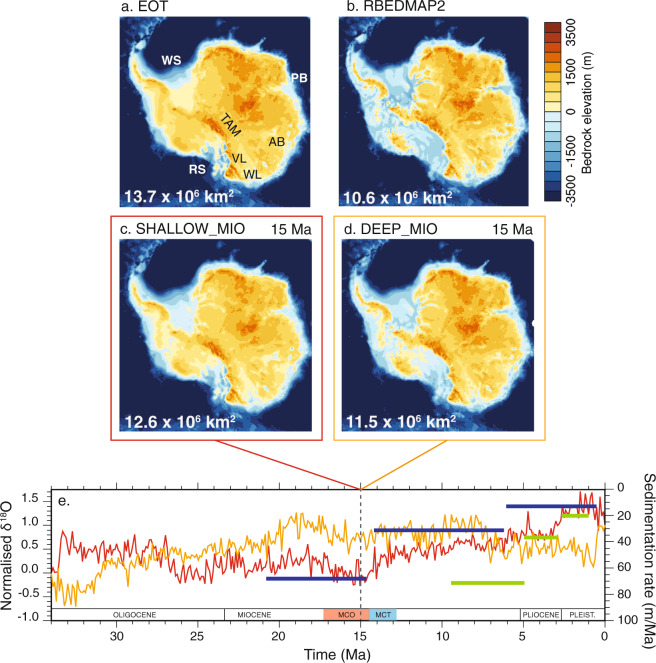
Topographies used as boundary conditions in the ice sheet simulations. (**a**) Eocene-Oligocene Antarctic maximum topography^6^ (EOT); (**b**) relaxed BEDMAP2 present-day topography^16,51^ (RBEDMAP2). (**c** and **d**) Mid-Miocene topographies interpolated from EOT and RBEDMAP2. Interpolation followed an index based on the normalized *δ*^18^O stack record^13^ ((**e**) red line) for the topography SHALLOW_MIO (**c**) assuming that most of the deepening of the WAIS and of the marine basins as well as filling and expansion of the circum Antarctica continental shelf occurred after the mid-Miocene Climatic Optimum (MCO). Sedimentation rates (m/Myr) from ODP sites 1165^14^ (blue lines) and 1095^15^ (green lines) are reported for comparison with *δ*^18^O record trend evolution. Sedimentation rates at both sites decrease while climate cools (increasing *δ*^18^O). The second mid-Miocene topography DEEP_MIO (**d**) was interpolated following the inverse evolution of global *δ*^18^O stack record (**(e)** orange line) assuming that most of the deepening of the WAIS and of the EAIS marine basins and filling and expansion of the circum Antarctic continental shelf occurred before the MCO. As a result, DEEP_MIO present deeper marine basins and continental shelf edge broader than in SHALLOW_MIO. See Methods for more details. The proportion of emerged topography is reported on each map (km^2^). Geographic locations used in the present study are also reported. RS: Ross Sea; WS: Weddell Sea; PB: Prydz Bay; AB: Aurora Basin; WL: Wilkes Land; VL: Victoria Land; TAM: Trans-Antarctic Mountains.

In the absence of pan-Antarctic paleo-topography and paleo-bathymetry reconstructions for the middle-Miocene, previous studies investigating the impact of past subglacial topography on AIS dynamics artificially increased or decreased elevation^2,3^. Partial regional reconstructions of the mid-Miocene glacial paleo-bathymetry exist though, for example for the Ross Sea^10^ or for the Weddell Sea^11^. Both reconstructions suggest that large progradation of the continental shelf occurred until about the mid-Miocene Climate Optimum (MCO, ≈17–14.3 Ma). Subsequent glaciations caused a widespread erosional event, documented in several locations around Antarctica, that led to the truncation of the stratal units that were deposited before the MCO. This event is dated around 16.7–14.2 Ma in the Ross Sea^12^ encompassing the entire MCO, and around 15 Ma in the Weddell Sea^11^. Those reconstructions (Supplementary Fig. S1) only exist for a limited spatial domain whereas pan-Antarctic paleo-topography and bathymetry are required as boundary conditions for ice sheet models. Furthermore, the age of those reconstructed mid-Miocene paleo-bathymetries is still uncertain. Following the philosophy of previous studies^2,3^, we use the global *δ*^18^O record^13^ that is consistent with the time evolution of sedimentation rates from deep drilling around the Antarctic margins (e.g. ODP leg 188 in Prydz Bay^14^ and ODP leg 178 in Antarctic Peninsula^15^) to derive two solutions of mid-Miocene topography and bathymetry at 15 Ma (Fig. 1a). These are based on an ice-free Eocene-Oligocene Antarctic reconstruction^6^ (EOT hereafter) and on the ice-free isostatically relaxed present-day topography and bathymetry BEDMAP2^16^ (RBEDMAP2 hereafter). See Methods for more details.

**Figure 2:**
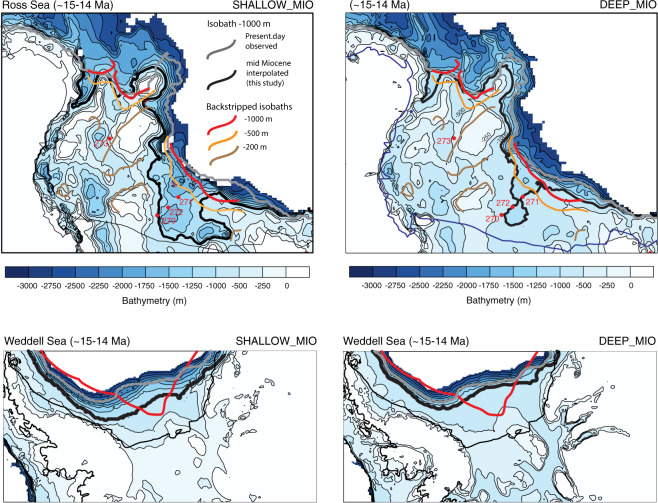
Blow-up of the Ross Sea and Weddell Sea interpolated mid-Miocene bathymetries SHALLOW_MIO and DEEP_MIO (see Fig. 1b,c and Methods). Black thick line corresponds to the −1000 meters isobath for the derived bathymetries while the grey thick line corresponds to present-day BEDMAP2 −1000 meters isobath. In the case of the Ross Sea, backstripped isobath^10,12^ −1000 meters, −500 meters and −200 meters from the Ross Sea Unconformity 4 (RSU4) from Fig. S1 are superimposed (see Supplementary informations). In the case of the Weddell Sea, the −1000 meter backstripped isobath is from a recent study^11^. DSDP sites 270, 271, 272 and 273 are also located. Paradoxically, in the Ross Sea, as in the other Antarctic marine basins, SHALLOW_MIO displays deeper bathymetry than in DEEP_MIO. “Shallow” here refers to the West Antarctic subglacial topography that is mostly emerged and thus shallower than in DEEP_MIO. Because the West Antarctic topography is mostly emerged in SHALLOW_MIO, the Ross-Sea and Weddell-Sea basins (and other marine basins of East Antarctica like the Wilkes Land, for example) are not yet filled by erosion and sediment transport as in DEEP_MIO, in which the West Antarctic topography is mostly below sea level (Fig. 1c,d).

The first mid-Miocene topography, SHALLOW_MIO, assumes that most of the deepening of WAIS and of East Antarctica (EAIS) marine basins, including the filling and expansion of the outer-most margins, occurs after the MCO (upper bound of the mid-Miocene glacial erosional event). It therefore still has very shallow bathymetry (most of West Antarctica is emerged) and relatively-smaller continental shelves at 15 Ma (Fig. 1c). Conversely, the second topography DEEP_MIO tests the hypothesis that part of the deepening of the WAIS and of the EAIS marine basins, as well as filling and expansion of the margins, occurs before the MCO (upper bound of the mid-Miocene glacial erosional event) and has deeper bathymetry and broader continental shelves than SHALLOW_MIO (Fig. 1d). As expected from geological evidence^17–19^, the comparison between reconstructed mid-Miocene paleo-bathymetry in the Weddell Sea^11^ and in the Ross Sea^10^ and our interpolated mid-Miocene bathymetries reveals that DEEP_MIO is in better agreement with data-based reconstructions than SHALLOW_MIO (Fig. 2). The MCO is probably a time with little erosion because the AIS extent is probably small and restricted to the inner continent. Substantial erosion of the continental shelf restarted from the MCT, when atmospheric CO_2_ started dropping again. In the following analysis, SHALLOW_MIO is considered as an earlier stage of continental shelf expansion relatively to DEEP_MIO. We use these four topographies (EOT, SHALLOW_MIO, DEEP_MIO, and RBEDMAP2) as boundary conditions for ice sheet simulations to test the impact of the gradual deepening and expansion of the continental shelf on the AIS dynamics and its response to mid-Miocene and late-Pleistocene glacial climate conditions as well as to different sub-ice-shelf melting and calving conditions.

## Numerical Experiments Design

Over the last 15 Myr, atmospheric CO_2_ concentration dropped^20,21^, leading to a gradual global climate cooling, marked by a major step in its evolution at the Plio-Pleistocene transition, about 3 Ma. Higher atmospheric CO_2_ concentration during mid-Miocene glacials (>280 ppm), combined with major oceanic and atmospheric circulation changes, led to mid-Miocene glaciations that were characterized by warmer and wetter conditions than during the late-Pleistocene glaciations when CO2 concentrations were significantly lower (<200 ppm) (Fig. S3). To explore the significance of these differences in climate and subglacial topography, ice sheet sensitivity simulations are performed for two end-member case studies, a mid-Miocene glaciation and a late-Pleistocene glaciation (here the Last Glacial Maximum, LGM), starting from their respective interglacial conditions.

Interglacial ice-sheet equilibrium spin-ups (150,000 model-years) are carried out for each of the four topographies. A first series of spin-ups is performed under mid-Miocene interglacial climate conditions^2^, characterized by a CO_2_ atmospheric concentration of 500 ppm and orbital parameters set for interglacial-like conditions (perihelion = June 21st; obliquity = 24.5°; eccentricity = 5%). A second series of spin-ups is computed using late-Pleistocene atmospheric forcing, in our case the last interglacial climate at 125 ka^22^ (CO_2_ = 273ppm; perihelion: July 23; obliquity:23.86°; eccentricity:4.2%). For each of the four topographies, the glacial advances of the AIS (equilibrium runs of 100,000 model-years) are branched on those initial interglacial spin-ups (ice volumes listed in Table S3). We use mid-Miocene glacial atmospheric forcing^2^ characterized by a CO_2_ atmospheric concentration of 280 ppm and orbital parameters set to glacial-like conditions (perihelion = December 21st; obliquity = 22.5°; eccentricity = 5%) and late-Pleistocene LGM climate^23^ (CO_2_ = 180 ppm; perihelion = Jan 4; obliquity = 22.91°; eccentricity = 1.8%) to force the ice-sheet simulations. Note that mid-Miocene and late-Pleistocene climate conditions have been generated with different climate models.

**Figure 3:**
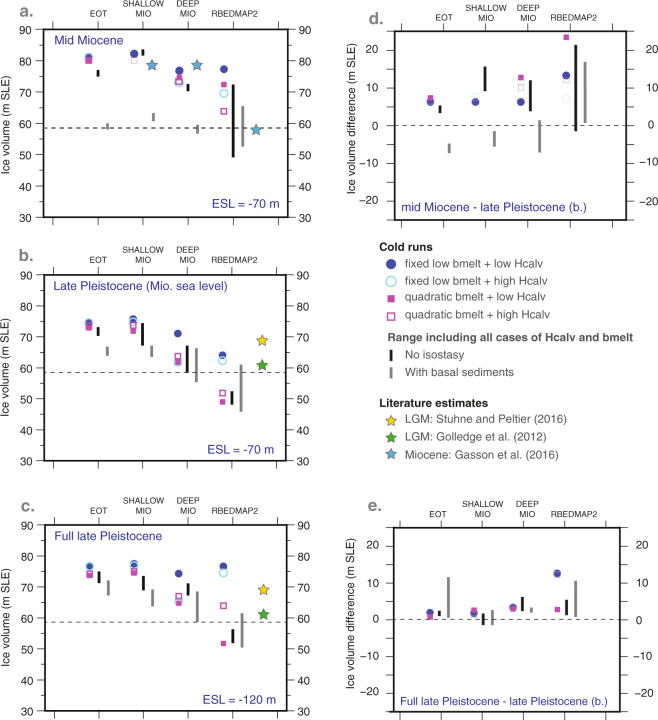
Simulated impact of subglacial topography and bathymetry on grounded ice volume response to different atmospheric forcing, oceanic forcing and eustatic sea levels. Ice sheet simulations are forced with (**a**) mid-Miocene glacial climate^2^, an eustatic sea level fall of 70 meters below present; (**b**) same as for a. but with late-Pleistocene glacial climate (here LGM^23^); (**c**) same as for (**b**) but with an eustatic sea level fall of 120 meters^29^. Thus, (**a** and **c**) corresponds to full mid-Miocene and full late-Pleistocene glaciation cases. The impact of atmospheric forcing on ice dynamics, i.e., the difference between (**a** and **b**) is shown in panel d. The impact of prescribed eustatic sea level on ice sheet dynamics, i.e., the difference between (**b** and **c**) is shown in panel e. For each of the four topographies, four simulations are carried out using different sub-ice-shelf melt rates (*bmelt*, blue dot and purple square) and calving thickness thresholds (*Hcalv*, blue circle and empty purple square). Simulations are repeated by inhibiting glacial isostasy (black bars) or by enhancing the impact of basal hydrology (grey bars). For these last two sets of simulations, only the range of volume spanned by the simulations is displayed. Individual ice volumes are reported in Tables S3–S5. Colored stars are simulated ice volume for LGM or Miocene from literature. Finally, dashed lines correspond to present-day Antarctic ice volume^51^. See Methods and Supplementary material for details on the settings of the model and simulations.

For each of the four topographies and starting from the warm spin-up simulations, four glacial simulations differing in the ocean-induced sub-ice-shelf melt rates and calving criterion (hereafter referred to as ocean forcing) are carried out (Fig. 3). In the first group of simulations (control, Table S3), ocean sub-ice-shelf melt rates are set to the spatially uniform value of 0.05 m/yr above 1000 meters depth and 1 m/yr below. Although some previous studies^24,25^ impose 0 m/yr in their glacial simulations, recent ocean modeling of the LGM^26^ shows that ocean water nearby the AIS margins is not at freezing point everywhere which is why we impose a minimal sub-ice-shelf melt rates over the continental shelf. Calving thickness thresholds are prescribed based on literature estimates varying between 150 meters^27^ and 200 meters^28^. These studies agree on the fact that the re-expansion of the AIS after a collapse is almost impossible if the calving threshold remains larger than 150 meters. Therefore, in our simulations we test intermediate ad-hoc calving threshold values of 120 meters and 160 meters (OF2, Table S3).

Recent idealized LGM ocean simulations using the present-day Antarctic configuration^26^ show that, despite global cold climate conditions, sub-ice-shelf melt rates in the vicinity of the Antarctic continental shelf break remain relatively high, close to present-day values, and penetration of warm Circum Polar Deep Water on the continental shelf occurs only in specific locations of the continental shelf edge. Therefore, in a second group of simulations (OF3 and OF4, Table S3 using the two different calving thresholds described above), a quadratic temperature-depth relationship on sub-ice-shelf melt rates is used^28^. These simulations yield very low melt rates for shallow depths and larger melt rates at the grounding line, which on average leads to a value of 0.06 m/yr, close to that imposed in the first group of simulations using OF1.

To disentangle the effect of climate from the effect of eustatic sea level change, three different sets of simulations (using the settings outlined in the preceding paragraph) are carried out: (1) mid-Miocene glaciation (with mid-Miocene glacial climate^2^) and eustatic sea level prescribed at 70 meters below present-day global mean sea level from a previous study^2^ (Fig. 3a); (2) full late-Pleistocene (with LGM climate^23^) and eustatic sea level set at −120 m (mean LGM sea level fall over various reconstructions^29^) (Fig. 3c); (3) a set of late-Pleistocene simulations using mid-Miocene glacial eustatic sea level of −70 m (Fig. 3b). Thereby, the comparison between the Miocene and the later set of simulations gives the impact of atmospheric forcing on the AIS dynamics (Fig. 3d), and the comparison between the full late Pleistocene and the later experiments provide the effect of eustatic sea level (Fig. 3e). Those simulations are considered as the standard simulations (with GIA but no prescribed basal sediments) throughout this study. All those simulations are repeated by inhibiting GIA to investigate its impact on the AIS dynamics in the various simulations. Simulations are carried out once again (with GIA) prescribing basal sediments and thus enabling the acceleration of ice flow if basal sediments are thick (see Supplementary Information). All simulations settings are detailed in Tables S1 and S2. In total, 180 simulations are carried out, varying sub-shelf melting, calving threshold, eustatic sea level and atmospheric forcing (surface air temperature and precipitation) for each topography. See Supplementary Informations for more details.

## Results

### AIS sensitivity to subglacial topography and ocean forcing

Our results show that the gradual deepening of the outer continental shelf from EOT to RBEDMAP2 induces an overall gradual decrease in ice volume along with increasing calving and sub-ice-shelf melting. This is particularly marked for DEEP_MIO and RBEDMAP2 in all three sets of simulations (Fig. 3). This decrease is independent of the atmospheric forcing or prescribed eustatic sea level. This is because a larger portion of the ice sheet is gradually exposed to the oceanic conditions (hereafter represented by sub-ice-shelf melting and calving) during the AIS advance on the continental shelf. However, the AIS dynamical behaviour differs between mid-Miocene and late-Pleistocene simulations. Sensitivity to ocean forcing increases notably when using RBEDMAP2 topography under mid-Miocene climate conditions. The large accumulation simulated in the Miocene experiments compensates for the ocean forcing, while this is not the case in the late-Pleistocene experiments.

In general, the Miocene wet climate leads to higher simulated ice volumes than under the dry late-Pleistocene glacial climate conditions (Fig. 3b,c). But as a consequence of our steady-state experiments, the LGM final ice volumes (despite lower accumulation rates) are comparable to that of the middle Miocene when using fixed low sub-ice-shelf melting rates. As a result of the difference in accumulation between the mid-Miocene and the late-Pleistocene simulations, the mechanisms causing the discrepancies in ice volume differ when using the most over-deepened bathymetry, i.e. RBEDMAP2. When an ice sheet is in dynamical equilibrium, ice fluxes through the grounding line partly compensate for sub-ice-shelf melting, dampening the thinning of the ice shelves. In fact, the mid-Miocene simulations exhibit a higher sensitivity to calving than to sub-ice-shelf melting (Fig. 3c). The opposite occurs in the late-Pleistocene simulations in which ocean basal melting prevails on calving because ice fluxes through the grounding line are weaker than in the mid-Miocene simulations (Fig. 3b). Finally when using RBEDMAP2, and with a sea level set at −70 m, the grounding line is located upstream to that simulated with a lower sea level (−120 m) because the ice sheet starts floating earlier than in the full late-Pleistocene simulations. Consequently, in the full late-Pleistocene simulations, the AIS can advance farther on the outer shelf and ice volumes are larger than when sea level is set to −70 m, but the AIS results more sensitive to oceanic conditions (Fig. 3b).

The capability of the AIS to anchor on bathymetric highs and pinning points is essential to its expansion. The weight of the ice sheet modulates the elevation of the bathymetric highs, and hence its ability to expand through glacio-isostatic adjustment (GIA). It has been recently shown that the fast rebound of the lithosphere in specific areas can partly compensate for the Marine Ice Sheet Instability by increasing the bed elevation, thereby providing new pinning points to the ice sheet^30,31^. In our ice sheet model, GIA follows an Elastic-Lithosphere-Relaxing-Astenosphere (ELRA) parameterization^32^, which is a simplified version of the fully self-gravitational GIA theory used in recent studies^30,31^. Nevertheless, the ELRA has been shown to satisfactorily capture the essential effect of GIA^32^ and is enough to illustrate the first order feedback between GIA, bathymetry and ice dynamics. In our mid-Miocene simulations, inhibiting GIA (black bars) yields lower ice volumes and reduces the sensitivity to ocean forcing (Fig. 3) because the AIS is not able to pin and expand. This is particularly striking for the late-Pleistocene simulations using RBEDMAP2 while in the mid-Miocene simulations, the large accumulation rates again allow the AIS to advance on the outer continental shelf. With an intermediate eustatic sea level, the ice sheet starts floating earlier than in the full late-Pleistocene simulations (Fig. 3b), which prevents the expansion of the AIS on the outer continental shelf and limits the impact of inhibiting GIA.

Finally, we examine the role of basal hydrology on the AIS dynamics. It has been observed that the presence of water -saturated soft sediments or basal meltwater at the base of the AIS can trigger an acceleration of the ice flow and induce large ice discharges to the ocean^33^. In our ice -sheet model, the presence of saturated sediments is used as a trigger to accelerate basal velocities (see Methods). As a result, in our mid-Miocene and late-Pleistocene simulations the acceleration of the ice flow leads to a smaller grounded ice volume (Fig. 3, grey bars). The largest discrepancies with the mid-Miocene and late-Pleistocene standard simulations occur when using mostly emerged topographies (EOT and SHALLOW_MIO). Basal hydrology indeed, applies only to the grounded part of the ice sheet and thus depends on the extent of the grounded area, which is likely to be larger over mostly emerged topographies. The main difference between mid-Miocene and late-Pleistocene simulations over EOT and SHALLOW_MIO resides in the extent of the area of saturated sediments. Relatively warm glacial mid-Miocene climate conditions yield thicker ice sheets, higher amount of basal meltwater and larger warm-based area than in the late-Pleistocene simulations (shown and discussed below). Because the ice flow over EOT and SHALLOW_MIO topographies is mostly driven by the basal hydrology conditions in both mid-Miocene and late-Pleistocene simulations, the sensitivity to ocean and atmospheric forcing is weaker than in the standard simulations. This holds for all cases.

### From Miocene to Pleistocene glaciations

**Figure 4:**
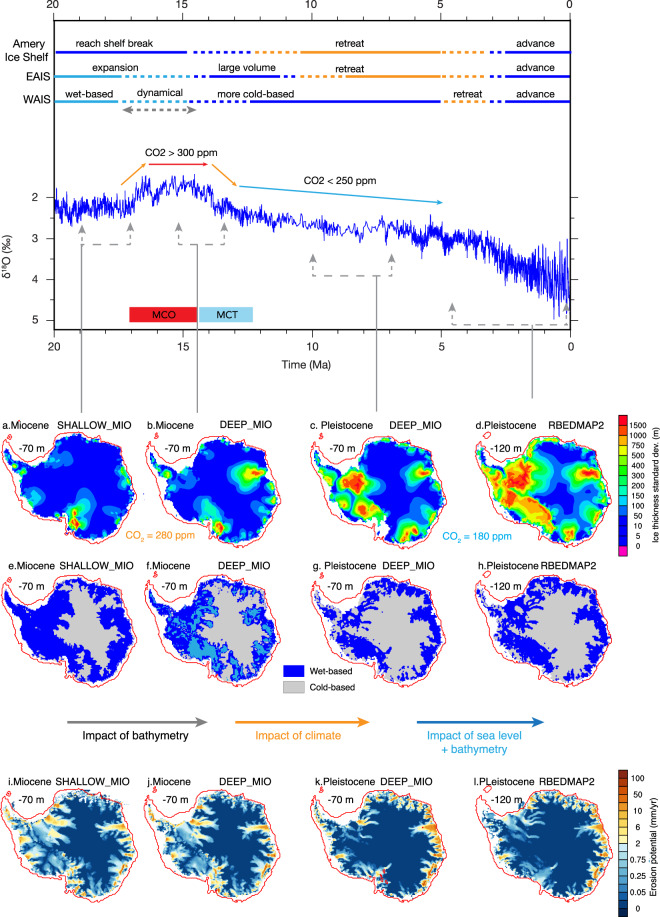
Top: AIS history adapted from various studies^17–19^; CO_2_ evolution^21^ and *δ*^18^O stack record^13^. Middle: Spatial sensitivity of grounded ice thickness to prescribed subglacial topography, atmospheric and ocean forcing (**a** to **d**) estimated by calculating the standard deviation of simulated ice thickness of the four ocean forcing cases (Table S2) over each indicated subglacial topography. Red thin lines correspond to the −1000 meters isobath of each subglacial topography. Frames (**e** to **h**) display the wet-based (dark blue) and cold-based (grey) areas for the control simulations (Table S2). On panel f, the areas where the simulated amount of basal meltwater is larger using DEEP_MIO than using SHALLOW_MIO subglacial topographies is shaded in light blue (dark blue in this case corresponds to areas where basal meltwater quantity is lower when using DEEP_MIO subglacial topography). Finally, bottom row shows the averaged erosion potential^52^ for the control simulations including the impact of basal hydrology (see Methods).

**Figure 5:**
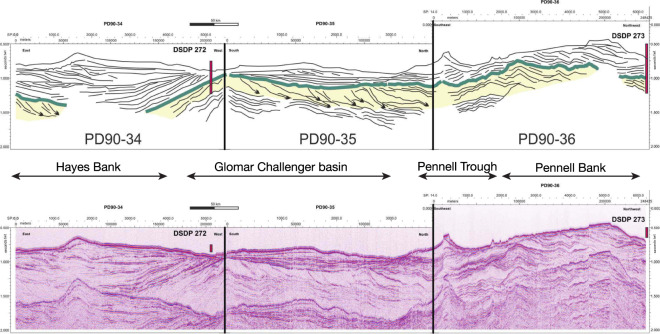
Seismic profile across the central Ross Sea showing the unconformity RSU4 (middle Miocene, green thick line on line drawing, top frame) truncating a thick progradational feature (yellow shade on line drawing, top frame), interpreted as glacial delta. The vertical scale is in two way travel time (in milliseconds). See supplementary Material and Fig. S1 for more information about RSU4. The thickness of the glacial delta is 250 meters assuming an acoustic waves travel time velocity of about 1700 m/s (based on stack velocity from the nearest multichannel seismic profiles). The glacial delta documents the expansion of ice sheet over the central Ross Sea continental shelf during the middle Miocene with large erosion rates and sediment transport, culminating with the subglacial surface RSU4, a basin-wide erosional event dated 16.7–14.2 Ma at DSDP sites 272 and 273^53,54^.

The analysis above aptly illustrates the feedback between topography, ice volume and ocean and atmospheric forcing. Using those conceptual simulations, we next attempt to draw a timeline for the various feedbacks in response to bathymetry changes in which SHALLOW_MIO can be considered as an earlier stage of the Miocene continental shelf evolution than DEEP_MIO. Likewise, RBEDMAP2 represents the last stages of the continental shelf aggradation during the Plio/Pleistocene simulations. Simulations performed over EOT are discarded from the following discussion. Our simulations show a gradual increase in the sensitivity to ocean and atmospheric forcing of the main marine basins of the WAIS and of the EAIS. This is supported by the geological interpretation of the long-term AIS evolution from the middle Miocene to the late Pleistocene (Fig. 4a–e):

(1) **Pre-MCO glacial stages** (Fig. 4a). SHALLOW_MIO is mostly emerged or shallow. As such, the AIS sensitivity to ocean forcing is low, except in Victoria Land where the bathymetry is deeper than in the Eastern Ross Sea (Fig. 1c) and thus exposes the ice sheet to ocean forcing more than in other sectors. Evidence from south Victoria Land^34^ suggests that this sector remained wet-based at least until the mid-Miocene Climatic Transition (MCT, ≈14 Ma Fig. 4). Recent seismic reflection analysis^35^ and ANDRILL sediment core records^19^ indicate large sediment supply to the western Ross Sea continental shelf until the late Miocene that is indicative of dynamic glacier activity. In our simulations, most of the AIS is wet-based, except in the central part of the EAIS (Fig. 4f), which promotes large erosion potential in the main ice streams areas of the EAIS and over the entire WAIS (Fig. 4k). Seismic reflection analysis of different transects across the eastern Ross Sea document large sediment transport from both EAIS and WAIS and fast progradation of the continental shelf until about 16.7–14.2 Ma, abruptly eroded by a subsequent intense glacial activity (Fig. 5). Our simulations over SHALLOW_MIO and under the mild mid-Miocene glacial climate conditions yield a thick wet-based WAIS (14 m sea level equivalent, SLE, Table 1), as inferred from geological evidence^18^. Similar to the Ross Sea, most of the Weddell Sea sector is also wet-based in our simulations, which also implies large erosion and transport of sediments to the continental shelf (Fig. 4k). This is compatible with the expansion of the continental shelf during the Miocene observed from seismic reflection analysis in both sectors^11^. In our simulations, the AIS is stable in Prydz Bay and reaches the continental shelf break, in agreement with observations^17^. Finally, in our simulations, the EAIS is massive as a result of the mild mid-Miocene climatic conditions (67 m SLE, Table 1). Over SHALLOW_MIO, in fact, the mean simulated AIS volume exceeds 80 meters SLE, about 22 m SLE larger than current AIS volume (Table 1).

**Table 1:** Simulated ice volumes (meters sea level equivalent, mSLE) averaged over the West Antarctic Ice Sheet (WAIS), over the Pacific sector of the East Antarctic Ice Sheet (EAIS_PACIFIC) and over the Atlantic sector of the EAIS (EAIS_ATLANTIC) for each of the subglacial topographies for the control simulations under glacial conditions.

Ice volumes (m SLE)	EOT	SHALLOW_MIO	DEEP_MIO	RBEDMAP2
**MIOCENE glaciation**				
WAIS	14.46	**14**.**27**	**13**.**15**	12.29
EAIS_PACIFIC	40.97	**41**.**61**	**38**.**33**	33.99
EAIS_ATLANTIC	25.30	**25**.**51**	**23**.**48**	24.37
TOTAL	80.6	**81**.**03**	**74**.**78**	70.75
**LGM (ESL** = −**70** **m)**				
WAIS	13.01	13.19	10.15	**7**.**00**
EAIS_PACIFIC	38.66	38.39	33.78	**31**.**21**
EAIS_ATLANTIC	22.26	22.16	20.61	**18**.**58**
TOTAL	73.93	73.95	64.55	**58**.**79**
**LGM (ESL** = −**120** **m)**				
WAIS	13.68	14.05	11.90	**11**.**90**
EAIS_PACIFIC	38.84	39.48	34.96	**33**.**48**
EAIS_ATLANTIC	22.75	22.64	20.98	**21**.**30**
TOTAL	75.29	76.17	67.84	**66**.**7**

Ice volumes correspond to the mean of the four simulations testing different oceanic forcing for each subglacial topography . The EAIS_PACIFIC includes the Amery Ice Shelf drainage basin, whereas the EAIS_ATLANTIC includes half of the Weddell Sea basin. Sectors are based on Antarctic drainage basins^ from Zwally *et al*.55^. Bold values correspond to the averaged volumes computed over the most realistic Miocene and Pleistocene subglacial topography and bathymetry, i.e., DEEP_MIO (according to Fig. 2) and RBEDMAP2, respectively.

(2) **First noticeable continental shelf filling and deepening around the MCO** (Fig. 4b). In DEEP_MIO, the main marine basins around Antarctica are slightly deeper and steeper than in SHALLOW_MIO, especially in Prydz Bay (Fig. 1d). Steeper slopes induce a slight inland retreat of the grounding line, in agreement with observations^17^. It also exposes a larger part of the ice sheet to the ocean, which amplifies the sensitivity to ocean conditions, increases the discharge, and yields lower simulated EAIS volumes (62 m SLE) than over SHALLOW_MIO. Deeper marine basins also enhance ice streams activity and as a result of elevated basal friction, the amount of basal meltwater increases compared to that simulated over SHALLOW_MIO (Fig. 4g). The reduction in sensitivity over the upstream part of Victoria Land results from changes in the bed elevation that probably provide topographic constraints to the ice flow. The deepening of the eastern Ross Sea leads to steeper slopes and thus to a more active WAIS in our simulations. Therefore, the sensitivity of the WAIS in the Ross Sea increases slightly compared to the simulations carried out over SHALLOW_MIO but mean ice volumes are still large (13 m SLE, Table 1). There is no noticeable expansion of the cold-based area in those simulations (Fig. 4g). This is because atmospheric forcing is similar to that prescribed over SHALLOW_MIO (geothermal heat fluxes are also identical in all the simulations). Consequently, erosion potential remains as high as that simulated over SHALLOW_MIO, although it is more focused in the marine basins in response to the deepening of the bathymetry in DEEP_MIO. Over this subglacial topography, the simulated mean AIS volume reaches about 75 meters SLE (Table 1).

(3) **Mid to late Miocene gradual climate cooling** (Fig. 4c). After the MCO, pCO_2_ drops below 250 ppm^21^. In the absence of a late Miocene simulation with low CO_2_ concentrations, we discuss our conceptual simulation carried out over DEEP_MIO, with mid-Miocene eustatic sea level (−70 m) but using the late-Pleistocene glacial atmospheric forcing with low CO_2_ (i.e., 180 ppm for LGM). Although synoptic circulation may have changed between late Miocene and late Pleistocene, the simulations illustrate the first order impact of low CO_2_ on the AIS dynamics over a bathymetry not as deep as today. With the cooling and drying of atmospheric forcing, the sensitivity of the entire WAIS, and that of the EAIS at Wilkes and Aurora Basins to ocean forcing increases substantially compared with previous steps (Fig. 4c). This highlights the interplay between accumulation and ocean forcing on the ice dynamics. With the lowering of CO_2_, climate dries and simulated low accumulation rates yield lower ice fluxes at the grounding line in general, which allows the ocean forcing to have a larger effect along the AIS margins. Recent analysis have shown that, indeed, after the MCO, Wilkes Land remained highly sensitive to ocean forcing^36^. Low air temperatures also induce a substantial expansion of the overall cold-based area especially below the WAIS (Fig. 4h). With the expansion of the cold-based area, the basal meltwater production occurs in the narrow valleys, further amplifying the impact of ocean forcing on the AIS dynamics. The AIS then is more prone to fluctuate on the outer continental shelf, further eroding the bed in the narrow valleys (Fig. 4k). As a consequence, erosion occurs more intensively along the EAIS margins while it largely diminishes below the WAIS and in the new cold-based areas. Simulated ice volume decreases over both the WAIS (to 10 m SLE, Table 1) and the EAIS (to 54 m SLE) compared to the mid-Miocene simulations.

(4) **Plio/Pleistocene glaciations** (Fig. 4e). With deeper but more expanded continental shelves and larger eustatic sea level drops (−120 m), the AIS is able to easily reach the continental shelf edge in the Weddell Sea and in the Ross Sea. Compared with previous simulations on shallower mid-Miocene bathymetries, with an overdeepened bathymetry such as RBEDMAP2, large sea level drops allow the AIS to fluctuate on a larger area of the outer continental shelf. As a result, the AIS is highly sensitive to ocean forcing, more than previously over the shallower mid-Miocene bathymetries. In this case, glacio-isostatic adjustment, combined with a larger sea level drop, is essential to the expansion of the AIS as shown previously (Fig. 3a). No further expansion of the cold-based area occurs relatively to the previous step (Fig. 4j) since prescribed atmospheric forcing are similar (Fig. 4c uses LGM climate forcing). As a result, erosion potential is highly similar to the one simulated in Fig. 4c. Because accumulation is low, the simulated AIS volume decreases to 66 m SLE (WAIS = 12 m SLE; EAIS = 55 m SLE) but is slightly larger than in the previous simulations. This is because in DEEP_MIO, the continental shelves are not as expanded as in RBEDMAP2, which limits the expansion of the AIS.

The expansion of the cold-based area, according to geological evidence might have started during the MCT^19^. Stratigraphic analysis from the western Ross Sea^35^, Aurora basin^37^ and from Wilkes Land^38^ document the turning point from a wet-based regime with a large sediment supply to a polar regime of the AIS with lower sediment supply during the late Miocene. Our simulations show a substantial spatial and quantitative reduction in erosion potential between the middle Miocene and the late Pleistocene along with AIS dynamical changes that possibly occurred between Fig. 4g,h. Results also suggest that mid-Miocene ice volumes might have been significantly larger as a result of wetter climate conditions during Mid-Miocene glaciations than during late-Pleistocene glaciations (Table S2), which is supported by geological evidence from the Transantarctic Mountains^34^ and from the ANDRILL sediment core records^19^. The main difference in ice volume between middle Miocene and late Pleistocene is computed over the Pacific sector of the EAIS (including Prydz Bay). Changes in seaway configurations from the Miocene to today (primarily the opening of the Bering Strait and the closure of Panama) might have induced changes in atmospheric and oceanic teleconnections originating in the Pacific and been responsible for moisture advection over the Pacific sector of Antarctica^39^.

## Conclusions

Our simulations clearly show that the evolution of the deepening and expansion of the continental margins influences the dynamical states of the AIS, independently from the atmospheric forcing (Fig. 3). Atmospheric forcing then modulates the magnitude of the AIS response to both bathymetry and ocean forcing. We suggest that by gradually eroding the continental margins, the AIS increased its own sensitivity to ocean forcing, which becomes a primary factor of AIS substantial variability during interglacials. We also suggest that this sensitivity is partly compensated by the large Pleistocene global atmospheric and oceanic cooling.

The interplay between atmospheric and oceanic conditions, sea level and bed morphology has a transient nature. In the present study, we used steady-state climate forcing to our ice-sheet simulations. It is likely that, in transient ice sheet simulations some compensation occurs between the atmospheric forcing and the oceanic sub-shelf melting that could have dampened the sensitivity to the various oceanic forcing tested in our study. Spatial resolution in our simulations is low, 40 km, and increasing resolution would probably induce different AIS dynamical behaviors since a more resolved subglacial topography would present different pining areas. Nevertheless, we maintain that our simulations show the primary mechanisms and interplay between the evolution of the subglacial topography, the deepening of continental shelf and the atmospheric and oceanic forcing. With these simulations, we show that the AIS contributed to the amplification of its own sensitivity to climatic conditions (atmosphere + ocean) by gradually eroding the continental margins, which likely changed its tipping points through time. The lack of past topographic reconstructions implies that we still have an imprecise idea of AIS response to past climate evolution, which is crucial to determine the AIS future evolution.

## Methods

### Interpolated mid-Miocene subglacial topographic maps

In the absence of numerous circum-Antarctic subglacial topographies and seabed reconstructions, previous studies investigating the impact of bed elevation prescribed artificial increase or decrease of elevation in specific sectors of the AIS^3^ or assumed a linear evolution of the bed elevation through time^2^. Here, we use the global *δ*^18^O stack^13^ to derive two solutions of mid-Miocene topography at 15 Ma (Fig. 1a) based on the Eocene-Oligocene Antarctic reconstruction^6^ (EOT) and the relaxed present-day subglacial topography BEDMAP2^16^ (RBEDMAP2). We assume that the *δ*^18^O evolution is representative of the glacial erosion and marine sedimentation occurring around Antarctica (Fig. 1, i.e., Leg 188 in Prydz Bay^14^ and Leg 178 in Antarctic Peninsula^15^). This implies that continental-margin expansion is representative of sedimentation rates (as a first-order approximation) but mostly bounded to climate evolution. The first mid-Miocene topography, i.e., SHALLOW_MIO, implies that most of the deepening occurs after the Miocene Climatic Optimum (MCO, ≈17–15 Ma) and thus signifies shallow bathymetry, i.e., mostly emerged topography more limited continental shelves than today (Fig. 1c):

1 $$SHALLOW\_MIO(t)=EOT+[(RBEDMAP2-EOT)\times \frac{(t-{t}_{34Ma})}{({t}_{0}-{t}_{34Ma})}]\times \alpha (t)$$

where *α*(*t*) is the normalized *δ*^18^O time serie such that *α*(*t*_34*Ma*_) = 0 and *α*(*t*_0_) = 1 (Fig. S2):

2 $$\alpha (t)=1-\frac{{\delta }^{18}O{(t)}_{norm}-{\delta }^{18}O{({t}_{0})}_{norm}}{{\delta }^{18}O{({t}_{34Ma})}_{norm}-{\delta }^{18}O{({t}_{0})}_{norm}}$$

At 15 Ma, *α*(*t*_15*Ma*_) = −0.10. Conversely, the second topography DEEP_MIO implies that part of the deepening happened before the MCO and exhibits broader continental shelves and deeper marine basins than SHALLOW_MIO (Fig. 1d):

3 $$DEEP\_MIO(t)=EOT+[(RBEDMAP2-EOT)\times \frac{(t-{t}_{34Ma})}{({t}_{0}-{t}_{34Ma})}]\times \alpha \_inv(t)$$

where the index *α_rev*(*t*) is reversed in time compared to the first subglacial topography solution such that *α_rev*(*t*) = *α*(*t*_34*Ma*_ − *t*). At 15 Ma, *α_rev*(*t*_15*Ma*_) = 0.65. We thus have four different topographies and bathymetries, EOT, SHALLOW_MIO, DEEP_MIO and RBEDMAP2, each representing successive gradual deepening of the continental shelves through time.

### Ice-sheet model

GRISLI is a 3D-thermo-mechanical ice sheet - ice stream - ice shelf model, able to simulate both grounded and floating ice^40,41^. The grounded part uses the Shallow Ice Approximation^42^ (SIA) whereas ice shelves and ice streams are treated using the Shallow Shelf Approximation^43^ (SSA). The ice shelf and ice stream formulation in GRISLI allows for a more realistic calculation of the growth of the ice sheet and ice shelves by allowing horizontal velocities to evolve freely and reach rapid basal sliding, particularly for the advance of ice onto the shallow continental shelves^44,45^. Isostasy is calculated by means of the Elastic-Lithosphere-Flexural-Asthenosphere model^32^ and seismic-based geothermal heat fluxes map is prescribed over the Antarctic domain^46^. To account for anisotropy in GRISLI, two enhancements factors are prescribed, one for grounded ice, handled with the SIA (*E*_*SIA*_), and one for ice shelves, handled with the SSA (*E*_*SSA*_). Usually, *E*_*SIA*_ is larger than 1, while *E*_*SSA*_ is lower than 1. In GRISLI, a simple basal hydrology scheme checks for the presence of both basal sediments and basal meltwater to trigger an acceleration of the ice flow by applying the SSA instead of the SIA approximation^44,47^. Our standard simulations do not include this effect. To test the impact of a basal sediment on the AIS dynamics, a basal sediments thickness map^48^ is prescribed as a boundary condition on the subglacial topography in an additional set of simulations. Calving occurs at the ice shelf front at a prescribed thickness *H*_*COUP*_ if the upstream ice flux cannot maintain the ice shelf balance. Basal melting under the ice shelves takes a prescribed uniform value that can vary with depth or follow a quadratic depth-temperature relationship following^27,49^. Atmospheric forcing is downscaled on the ice-sheet model grid and corrected by means of a standard lapse rate (6.5 °C) and precipitation correction factor (0.07% C^−1^). The ablation is calculated using the semi-empirical Positive Degree Day method^50^ (PDD) and up to 60% of the surface melt is able to refreeze. Surface mass balance is kept fixed for the entire simulations. The ice-sheet model is run on a 40-km horizontal cartesian rectangular regular grid using the polar stereographic projection (71°S as standard parallel). Unless otherwise stated, we use the default parameters listed in Supplementary Table S1.

## Electronic supplementary material

Supplementary Information
