# Author Correction: Past continental shelf evolution increased Antarctic ice sheet sensitivity to climatic conditions

**DOI:** 10.1038/s41598-020-67554-w

**Published:** 2020-06-25

**Authors:** Florence Colleoni, Laura De Santis, Enea Montoli, Elisabetta Olivo, Christopher C. Sorlien, Philip J. Bart, Edward G. W. Gasson, Andrea Bergamasco, Chiara Sauli, Nigel Wardell, Stefano Prato

**Affiliations:** 1 Fondazione Centro Euro-Mediterraneo Sui Cambiamenti Climatici, 40128 Bologna, Italy; 2 Istituto Nazionale di Oceanografia Sperimentale, 34010 Sgonico, Italy; 3 Dipartimento Scienze Fisiche, della Terra e dell’Ambiente, Universita di Siena, 53100 Siena, Italy; 4 Earth Research Institute, University of California, Santa Barbara, California 93106 USA; 5 Louisiana State University, Department of Geology and Geophysics, Baton Rouge, Louisiana 70803 USA; 6 Department of Geography, University of Sheffield, Sheffield, S10 2TN UK; 7 CNR-ISMAR, Arsenale Tesa 104, Castello 2737/F, 30122 Venice, Italy; 8 A.P.E. Research srl, Area Science Park, Basovizza Campus, 34149 Trieste, Italy

Correction to: *Scientific Reports* 10.1038/s41598-018-29718-7, published online on 27 July 2018

The Acknowledgements section in this Article is incomplete.

“This work is supported by the PNRA national Italian project PNRA16_00016, “West Antarctic Ice Sheet History from Slope Processes – Eastern Ross Sea (WHISPERS)” and by the MAE bilateral Italy-US project US16GR04, “Global Sea Level Rise & Antarctic Ice Sheet Stability predictions: guessing future by learning from past (GSLAISS)”. We acknowledge the CMCC Foundation for providing computing resources. This research is a contribution to the SCAR PAIS program (https://www.scar.org/science/pais/).”

should read:

“This work is supported by OGS and CINECA under HPC-TRES Program Award Number 2018-01, by the PNRA national Italian projects PNRA16_00016 WHISPERS and PNRA16_00293 GLEVORS, and by the MAE bilateral Italy-US Project US16GR04, “Global Sea Level Rise & Antarctic Ice Sheet Stability predictions: guessing future by learning from past (GSLAISS)”. We acknowledge the CMCC Foundation for providing computing resources. We acknowledge the Antarctic Seismic Data Library (https://sdls.ogs.trieste.it) for providing open access to international marine seismic data. This research is a contribution to the SCAR PAIS program (https://www.scar.org/science/pais/).”

