# Supplementary material for: Past continental shelf evolution increased Antarctic ice sheet sensitivity to climatic conditions
Source: Sci Rep. 2018 Jul 27;8:11323. doi: 10.1038/s41598-018-29718-7 (PMC6063862; doi:10.1038/s41598-018-29718-7)
Supplement: Supplementary file 1 — Supplementary Information [file 41598_2018_29718_MOESM1_ESM.pdf]

Past continental margins evolution increased Antarctic ice sheet sensitivity to climate conditions

Florence Colleoni[1,*], Laura De Santis[2], Enea Montoli[1], Elisabetta Olivo[2,3], Christopher C. Sorlien[4], Philip J. Bart[5], Edward G. W. Gasson[6], Andrea Bergamasco[7], Chiara Sauli[2], Nigel Wardell[2], and Stefano Prato[8]

[1]Fondazione Centro Euro-Mediterraneo sui Cambiamenti Climatici, Bologna, 40128, Italy
[2]Istituto Nazionale di Oceanografia Sperimentale, Sgonico, 34010, Italy
[3]Dipartimento Scienze Fisiche, della Terra e dell'Ambiente, Universit di Siena, 53100, Siena, Italy
[4]Earth Research Institute, University of California, Santa Barbara, Santa Barbara, California, 93106, USA
[5]Louisiana State University, Department of Geology and Geophysics, Baton Rouge, Louisiana 70803, USA
[6]Department of Geography, University of Sheffield, Sheffield, S10 2TN, UK
[7]CNR-ISMAR, Arsenale Tesa 104, Castello 2737/F, Venice, 30122, Italy
[8]A.P.E. Research srl, Area Science Park, Basovizza Campus, Trieste, 34149, Italy
[*]Corresponding author: Florence Colleoni, flocolleoni@gmail.com

June 8, 2018

List of supplementary informations

- Ice sheet model basal hydrology
- Ice sheet model limitations
- Ross Sea Unconformity 4 (RSU4) paleodepths backstripping procedure
- Figure S1: RSU4 backstripped map
- Figure S2: Air temperature and accumulation difference between Miocene cold climate and LGM
- Figure S3: Simulated total grounded ice volume difference between Miocene and Pleistocene simulations.
- Table S1: GRISLI main model parameters
- Table S2: Ice-sheet simulation settings
- Table S3: Simulated ice volumes for the Miocene glacial simulations (Figure 2a)
- Table S4: Simulated ice volumes for the full late Pleisotcene glacial simulations (-120 m) (Figure 2c)
- Table S5: Simulated ice volumes for the late Pleistocene glacial simulations and Miocene sea level (-70 m) (Figure 2b)

Ice-sheet model basal hydrology

The role of basal hydrology in the AIS dynamics is essential. Measurements and observations show that subglacial lakes exist below the ice and that they are potentially connected by mean of a hydrological network of channel (Wright and Siegert, 2011; Wright et al., 2012). The accumulation of basal meltwater and saturated soft sediments at the base of the AIS can trigger ice flow acceleration and induce large ice discharges to the ocean (Stearns et al., 2008). In GRISLI, a simple basal hydrology scheme checks for the presence of both basal sediments and basal meltwater to trigger an acceleration of the ice flow by applying the SSA instead of the SIA approximation (Peyaud, 2006; Alvarez-Solas et al., 2011). Previous works (Alvarez-Solas et al., 2011) focusing on the response of the Laurentide ice sheet to ocean warming during Heinrich events have shown that this combination between basal hydrology and SSA is able to capture the fast retreat of the grounding line and generate ice discharges compatible with observed IRD rates.

Ice-sheet model limitations

The position of the grounding line is determined according to a flotation criterion. As in many other ice-sheet models, grounding line position is calculated based on the balance of flux through the grounding-floating transition, which does not allow grounding-line fluxes to fully capture the dynamic nature of grounding-line migration in the way that higher-order or full Stokes models do. To optimise the grounding line dynamics in SIA/SSA models, spatial resolution is crucial and should at least reach 5 km (Pattyn et al., 2013). However, running long-term paleo-ice sheet simulations with such models at such resolution is still not doable. To run the large number of simulations presented in this study, almost 200, over the entire Antarctic domain, we choose a 40-km grid. Golledge et al. (2015), who use the PISM SIA/SSA model with slightly improved grounding line dynamics compared with GRISLI, show that the difference between 40 km and 5 km grid in both extent and volume is very small (see their extended Figure 6). Our study does not aim at reproducing the geographical extent of the AIS accurately but focuses on equilibrium simulations and sensitivity experiments in which changes occur on long-term and on large spatial scale in response to large changes in the bathymetry or subglacial topography.

The GRISLI ice-sheet model calculates surface melt by means of the PDD semi-empirical method. This methods has been calibrated based on measurements performed in Greenland. Melting computation is only based on surface air temperature changes and does not account for changes in surface albedo. In addition, the snowpack is represented by a single snow layer and the maximum amount of refreezing is prescribed. Previous studies have shown that the use of the PDD method tends to amplify surface melt under warm climate conditions (Van de Berg et al., 2011) and refreezing processes are over-simplified compared to a proper energy balance model (Bougamont et al., 2007). However, under cold conditions, the PDD provides satisfying results and many paleoclimate simulations are still carried out with ice-sheet model using the PDD method such as the recent contribution by Gasson et al. (2016) about the Antarctic dynamics under Miocene climate conditions or the study by Petrini et al. (2018) about the last deglaciation in Eurasia. In fact, the use of a proper energy balance requires the regular update of surface atmospheric circulation in function of ice-sheet elevation changes, which is prohibitive in the case of long-term simulations. Comparison between our simulated LGM (volume and extent) and mid Miocene ice volumes and those from previous studies based on different physics of the grounding line or with finer spatial resolution, shows that GRISLI performs very well (Figure 3A and 3C), which ensures that the results obtained in this study are reasonable. We thus believe that the conclusions of this study are robust and would be fairly similar on higher spatial resolution grid.

Ross Sea Unconformity 4 paleodepths backstripping procedure

Since the Miocene, continental margins around Antarctica have expanded and deepened as a results of marine sedimentation and glacial erosion. Miocene bathymetry is consequently buried under younger sedimentary

sequences. To retrieve mid-Miocene continental shelf paleodepths and morphology of the Ross Sea, post-rift backstripping modelling has been applied to depth contour map of the Ross Sea Unconformity 4 (RSU4), previously compiled in the framework of the ANTOSTRAT project (Cooper et al., 1995). **Figure S1** shows the distribution of the RSU4 in the central and eastern Ross Sea that was not affected by Cenozoic tectonics, that is mainly confined in the western Ross Sea. An update of the RSU4 ANTOSTRAT map is ongoing (IPY-Rossmap and MAE-GSLAISS Projects) and it mainly implies the inclusion of the continental slope and rise and in some coastal areas of the western Ross Sea where RSU4 can be traced on seismic data collected after 1995. The ANTOSTRAT RSU4 paleodepths map has been restored after removing sediment load of sequences above RSU4, and decompacting the sediments below RSU4, up to the basement, using the backstripping technique (Steckler and Watts, 1978; Kusznir et al., 1996; Roberts et al., 1998). RSU4 paleodepths map was constrained by lithological, density and porosity from DSDP leg 28 drilling sites (DSDP 270, 272 and 273, Hayes and Frakes, 1975). The approach and parameter values used for the backstripping follow De Santis et al. (1999). The glaciomarine sequence at DSDP sites has an averaged grain density (ρ_g) of 2.5 g/cm^3, a modern averaged saturated sediment density (ρ_s) of 2.0 g/cm^3, and a modern averaged porosity of 0.4 (Barrett and Froggatt, 1978).The same porosity and grain densities have been found at similar depths in the glaciomarine sediments retrieved at Cape Roberts sites, close to McMurdo Sound area (Team, 1998, 1999, 2000) and at ANDRILL sites (Niessen et al., 2007; Dunbar et al., 2007, 2009). A depth-porosity exponential relationship ($\phi(\mathrm{z}) = \phi_0 \mathrm{e}^{-cz}$) using a surface porosity (ϕ_0) of 0.45 and a compaction coefficient (c) of 0.45 km^{-1} obtained from stack and refraction velocities in the Eastern Basin, was used to backstrip the Ross Sea sequences following the approach of (De Santis et al., 1999).

Removing sediment and water loads above RSU4 induces an isostatic adjustment of the paleobathymetry that can be calculated assuming a local Airy isostasy or a flexural isostasy. The main difference between the two compensations resides in the small lithospheric Effective Elastic Thickness (EET) prescribed in the flexural model (e.g. 5 -10 km), although this value increases with increasing lithosphere rigidity (De Santis et al., 1999). Figure S2 that shows an east-west section across the Ross Sea obtained by the seismic section BGR80-002 intersecting DSDP site 272, illustrates the difference in RSU4 paleodepths restoration, assuming different EET values. In the Ross Sea, EET values ranging from 30 to 60 km have been calculated by (Tesauro et al., 2012) while (Wilson et al., 2012) used a spatially uniform value of 35 km to retrieve the Eocene/Oligocene transition pan-Antarctic topography and bathymetry. For example, using EET=0 or 45 km induces a depth difference of about 100 m at site DSDP 272. and a shallower depth in the near Central High. Therefore, the continental shelf at the time of RSU4 may have been up to 100 m shallower than the one shown in **Figure 2**, reconstructed assuming Airy local isostatic compensation.

The foraminifer assemblage and the overall sedimentary facies at site DSDP 272 would suggest deposition in relatively deep water ($>$ 200-500 m) in an ice distal environment at the time of deposition of sediment above RSU4, shallowing upward to a more ice proximal environment (Hayes and Frakes, 1975; Steinhauff and Webb, 1987). This is consistent with the paleodepth reconstructed after backstripping the RSU4 map. The sedimentary section and the seismic facies at DSDP site 272 show the occurrence of a grounding zone wedge downlaping on RSU4 between 14.3 and 13.8 Ma (Steinhauff and Webb, 1987; De Santis et al., 1997), suggesting ice expansion over the eastern Ross Sea in the late Miocene. The contribution of post-rift thermal subsidence is based on a McKenzie model, and assumes a rift age of 76 Ma (Wilson and Luyendyk, 2009), with an age for extension in the eastern Ross Sea dated 90-100 Ma (Fitzgerald and Baldwin, 1997; Luyendyk et al., 2003; Siddoway et al., 2004). Associated crustal stretching adopts the spatially variable stretching coefficient (β) ranging from 1.8 to 2.5 over most of the eastern Basin and Central High, and calculated from (Busetti et al., 1999).

The depth conversion of the RSU4 map displayed in Figure S1 has been obtained by using the stack seismic reflection velocities and, where available, the seismic refraction velocities (see AGU Atlas (Cochrane et al., 1995). New seismic data have been recently acquired in the southernmost Ross Sea, where RSU4 is eroded and older strata outcrop at the sea bed (Sorlien et al., 2007). New seismic surveys have also been recently carried out in the coastal areas of the western Ross Sea (Pekar et al., 2013; Henrys et al., 2000; Sauli et al., 2014) and in continental slope of the central (Kim et al., 2018), western (Granot et al., 2010) and eastern Ross Sea (Lindeque et al., 2016; De Santis et al., 1997). These data will be used to broaden

the current RSU4 map in a future and more specific contribution. However, the new data will not affect the paleodepth restoration of the central and eastern Ross Sea continental shelf presented here and previously published by (Brancolini et al., 1995) because the depth of the RSU4 will not be changed here, since it was defined and mapped on the basis of seismic and stratigraphic information from the drill sites that remain valid. Recent drill sites analysis (Naish et al., 2008; Harwood et al., 2008; McKay et al., 2017) will allow to revise and refine the age and paleodepth estimate of the ANTOSTRAT RSU4 map (on-going work by the authors), so far ranging from 16.8 – 14.7 Ma. Nevertheless, the restored RSU4 paleobathymetry based on ANTOSTRAT compilation and displayed in Figure S1 is still robust and can be used to qualitatively compare with the bathymetries used in the present study and support the conclusions.

Figure S1

Unpublished restored Mid-Miocene paleodepths from RSU4 backstripping calculations follows De Santis et al. (1995) and De Santis et al. (1999). Drill sites used to constrain the backstripping calculations are plotted on the map. White empty areas indicate where seismic reflection data are missing and thus backstripping of the RUS4 is not possible. Seismic trace of profiles displayed in Figure 5 of the main manuscript is reported on the map. Coastlines are from IBCSO (Arndt et al., 2013).

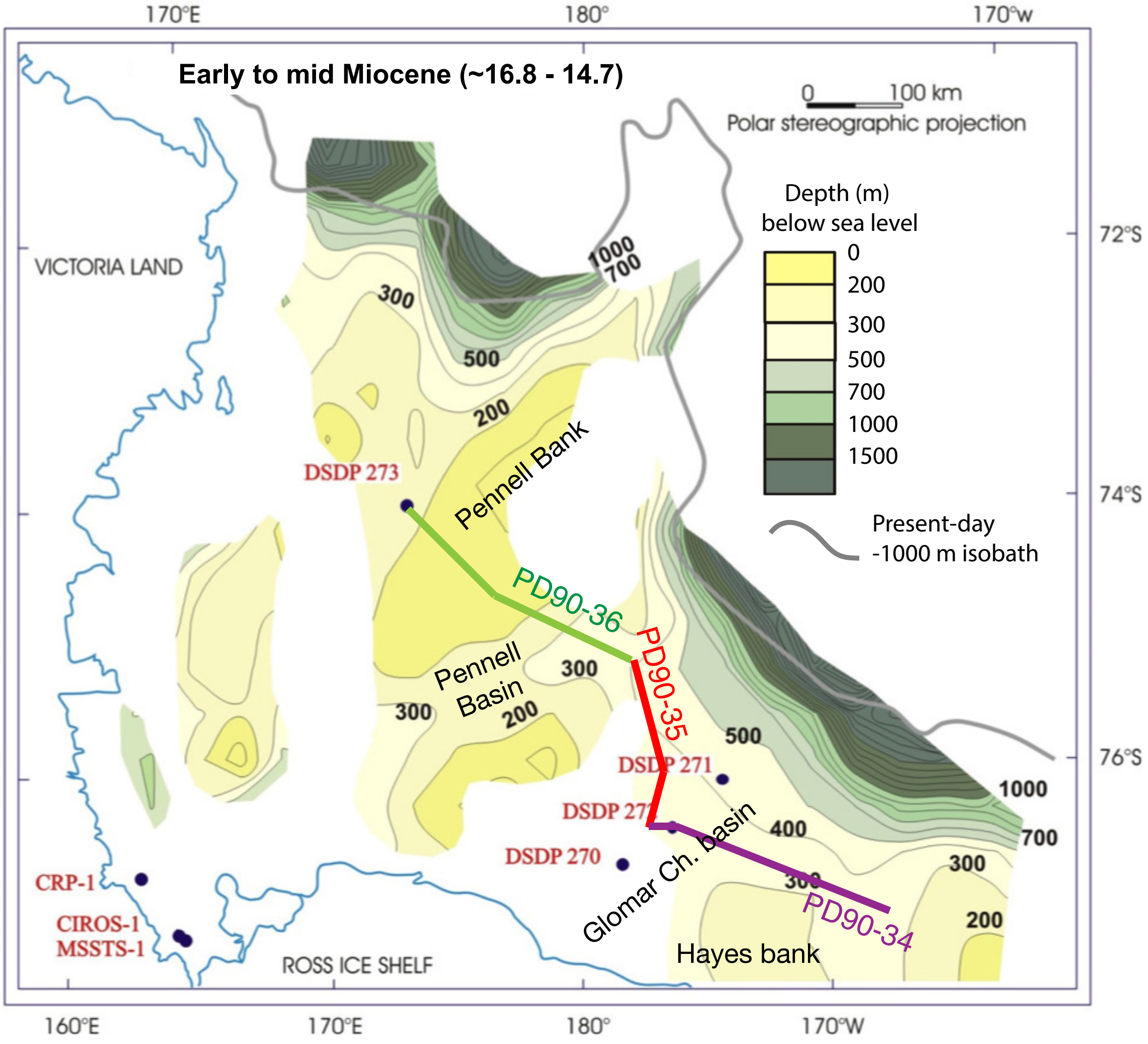

Figure S2

Simulated Mid-Miocene glacial atmospheric mean annual and summer (December-January-February, DJF) surface air temperatures in °C (a. and b.) and mean annual accumulation in m/yr (c.) from Gasson et al. (2016). Comparison between Mid-Miocene glacial and LGM atmospheric forcing (d. to f.). Note that we display the accumulation ratio (dimentionless), i.e. Mid-Miocene/LGM. Simulated LGM atmospheric forcing (g. to i.) are from Brady et al. (2013).

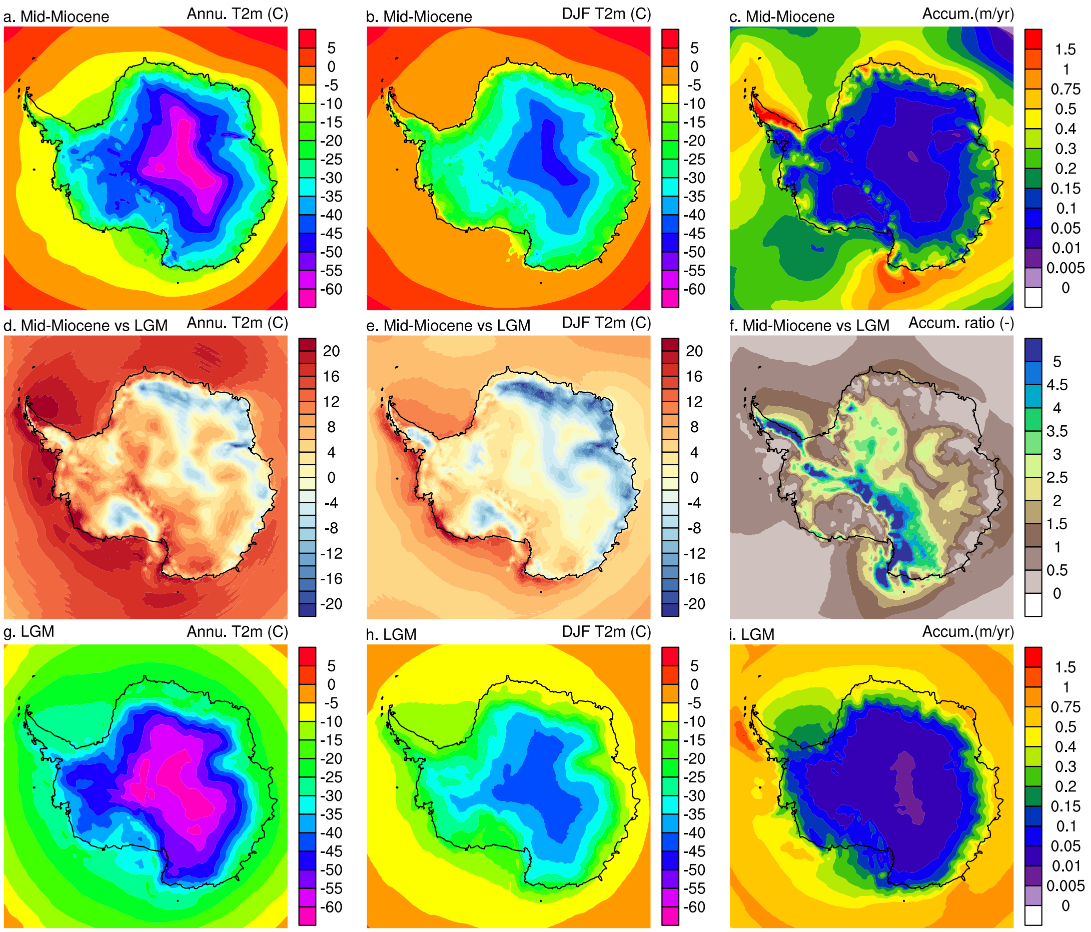

Table S1

Standards model parameters used by GRISLI in the simulations.

Parameter	Default value	Unit	Description
**Enhancement factors**			
E_3^{SIA}	3	dimensionless	SIA enhancement factor, Glen
E_1^{SIA}	1	dimensionless	SIA enhancement factor, linear
E_3^{SSA}	0.37	dimensionless	SSA enhancement factor, Glen
E_1^{SSA}	0.37	dimensionless	SSA enhancement factor, linear
T_3^{trans}	-6.5	$^\circ$C	Transition temperature of deformation, Glen
Q_3^{cold}	$7.820 \cdot 10^4$	$J \cdot mol^{-1}$	Activation energy below transition, Glen
Q_3^{warm}	$9.545 \cdot 10^4$	$J \cdot mol^{-1}$	Activation energy above transition, Glen
T_1^{trans}	-10	$^\circ$C	Transition temperature of deformation, linear
Q_1^{cold}	$4.0 \cdot 10^4$	$J \cdot mol^{-1}$	Activation energy below transition, linear
Q_1^{warm}	$6.0 \cdot 10^4$	$J \cdot mol^{-1}$	Activation energy above transition, linear
**Climate forcing**			
λ	Krinner and Genthon (1999)	$^\circ$C/km	elevation-dependent lapse rate
γ	5	%/C$^\circ$	Precipitation correction factor
**PDD parameters**			
C_{ice}/C_{snow}	0.008 / 0.003	mm/d/$^\circ$C	Melting coefficients for ice and snow
σ	5	$^\circ$C	Standard deviation of daily air temperature
csi	60	%	Percentage of refreezing
**Ice streams parameters**			
sediment map	Map of sediment thickness	m	Laske and Masters (1997)
h_w^*	250	m	Critical hydraulic head
c_f	$2 \cdot 10^{-5}$	dimensionless	Basal dragging coefficient
**Ice shelves parameters**			
H_{calv}	120; 160; 180	m	Critical thickness of ice-shelf front
b_{melt}	0.05/1	m/yr	Fixed sub-shelf Melting rate
	Quadratic melt-depth relationship	m/yr	DeConto and Pollard (2016)
**Solid Earth**			
Isostasy	Lemeur and Huybrechts (1996)		ELRA
τ_r	3000	yrs	Characteristic relaxation time
GHF	Maule et al. (2005)	mW/m^2	Geothermal heat flux

Table S2

Table S2: Simulation settings: four cases of ocean forcing and calving settings used in the simulations and applied to each different bedrocks (see Methods and **Table S1**). In OF1 and OF2, sub-shelf melting on the shallow continental shelves up to -1000 m depth is fixed to 0.05 m/yr following (Obase et al., 2017) and 1 m/yr at higher depth on the continental slopes and abyssal plains to account for the presence of circum-Antarctic warm waters. PD refers to the sub-shelf melting parameterization from DeConto and Pollard (2016) tuned on present-day sea surface temperature values, to which 1°C is removed to mimic glacial sea surface temperatures. This preserves the quadratic depth-temperature relationship and yield no sub-shelf melt for shallow depths and high melting rates at the grounding lines such that the averaged melt rates remain similar to the prescribed in OF1 and OF2. Prescribed eustatic sea level is indicated in brackets. The difference between Miocene and LGM simulations gives the impact of atmospheric forcing on the AIS dynamics, the difference between Late Pleistocene and Full Late Pleistocene simulations gives the impact of sea level on the AIS dynamics. Those simulations are run for each of the four bedrocks displayed in Figure 2 and are referred to as the standard simulations.

**Simulations Settings**	**Atmospheric forcing**	**Sub-shelf melt**	**Calving**
ID		$b_{melt}(m/yr)$	H_{coup} (m)
**Control**	Miocene (-70 m)	0.05 to 1	120
**OF2**	"	0.05 -1	160
**OF3**	"	PD - 1°C	160
**OF4**	"	PD - 1°C	160
**Control**	Late Pleistocene (-70 m)	0.05 to 1	120
**OF2**	"	0.05 to 1	160
**OF3**	"	PD - 1°C	160
**OF4**	"	PD - 1°C	160
**Control**	Full Late Pleistocene (-120 m)	0.05 to 1	120
**OF2**	"	0.05 to 1	160
**OF3**	"	PD - 1°C	160
**OF4**	"	PD - 1°C	160

Table S3

Table S3: Simulated spin-up interglacial ice volumes (meters sea level equivalent, mSLE) averaged over the West Antarctic Ice Sheet (WAIS), over the Pacific sector of the East Antarctic Ice Sheet (EAIS_PACIFIC) and over the Atlantic sector of the EAIS (EAIS_ATLANTIC) for each of the bedrock solution for the standard simulations under glacial conditions (top) and interglacial conditions (bottom). Ice volumes correspond to the mean of the four simulations testing different oceanic and calving forcing for each bedrock solution. The EAIS_PACIFIC includes the Amery Ice shelf drainage basin, the EAIS_ATLANTIC includes half of the Weddell Sea basin. Sectors are based on Zwally et al. (2012) drainage basins. Bold ice volume values corresponds to the most realistic cases of Miocene and Pleistocene, i.e. over the interpolated Miocene bedrock and RBEDMAP2, respectively. More details are provided in the Methods.

**MIOCENE interglacial**				
**WAIS**	1.32	**0.96**	**0.63**	0.40
**EAIS_PACIFIC**	28.35	**27.28**	**25.92**	21.58
**EAIS_ATLANTIC**	16.33	**14.86**	**13.93**	13.29
**TOTAL**	46.00	**43.10**	**40.49**	35.27
**MIS5 (ESL = -70 m)**				
**WAIS**	11.70	11.30	1.85	**5.64**
**EAIS_PACIFIC**	37.15	37.12	27.55	**31.23**
**EAIS_ATLANTIC**	22.24	21.44	15.80	**15.90**
**TOTAL**	71.09	69.85	45.20	**52.78**
**MIS5 (ESL = -120 m)**				
**WAIS**	11.41	11.77	1.84	**4.50**
**EAIS_PACIFIC**	37.28	37.29	27.22	**31.09**
**EAIS_ATLANTIC**	22.11	21.60	15.93	**16.00**
**TOTAL**	70.79	76.60	46.12	**55.63**

Table S4, S5, S6

Table S4: Simulated Miocene glacial ice volumes (meters sea level equivalent, mSLE) for all simulations displayed in Figure 3c. See Table S3 for WAIS, Pacific sector and Atlantic sector of EAIS individual ice volumes. b_{melt} is the sub-shelf melting set at fixed values (0.05 m/yr above 2000 meters and to 1m/yr below this depth) or calculated by means of the depth-temperature quadratic relationship from DeConto and Pollard (2016, PD hereafter). To simulate glacial-like sub-shelf melt rates, we removed 1°C to the prescribed modern temperature in the parametrisation of DeConto and Pollard (2016).H_{coup} corresponds to the ice shelf from thickness threshold for calving. Finally, EOT, SHALLOW_MIO, DEEP_MIO and RBEDMAP2 corresponds to the four bedrocks used as boundary conditions to the ice sheet simulations and displayed in Figure 3.

Table S5 displays the simulated ice volume for the full LGM experiments plotted in Figure 3c.

Table S6 displays the simulated ice volume for the LGM experiments in which eustatic sea level is prescribed to mid Miocene value of 70 mbsl and plotted in Figure 3b.

Mid-Miocene	Settings b_{melt}; H_{coup}	WAIS	EAIS_PACIFIC	EAIS_ATLANTIC	TOTAL
**Standard simulations**					
**EOT**	0.05 to 1 m/yr ; 120 m	14.80	41.43	25.25	80.98
**SHALLOW_MIO**	"	14.54	42.08	25.28	81.90
**DEEP_MIO**	"	13.46	40.06	23.69	77.21
**RBEDMAP2**	"	14.25	37.78	24.85	77.28
**EOT**	0.05 to 1 m/yr ; **160 m**	14.42	41.45	25.27	81.14
**SHALLOW_MIO**	"	14.06	42.44	25.80	82.30
**DEEP_MIO**	"	12.97	37.35	23.15	73.47
**RBEDMAP2**	"	11.59	33.58	24.31	69.48
**EOT**	**PD - 1°C**; 160 m	14.25	40.49	25.35	80.08
**SHALLOW_MIO**	"	14.22	40.30	25.44	79.97
**DEEP_MIO**	"	12.78	37.58	23.50	73.86
**RBEDMAP2**	"	9.17	31.15	23.55	63.87
**EOT**	PD - 1°C; **120 m**	14.37	40.51	25.32	80.20
**SHALLOW_MIO**	"	14.17	40.99	25.43	80.59
**DEEP_MIO**	"	13.40	37.58	23.59	74.57
**RBEDMAP2**	"	14.15	33.44	24.79	72.39
**No GIA**					
**EOT**	0.05 to 1 m/yr ; 120 m	13.96	38.43	23.58	75.98
**SHALLOW_MIO**	"	14.14	42.83	26.14	83.11
**DEEP_MIO**	"	12.97	36.97	21.87	71.81
**RBEDMAP2**	"	12.98	34.07	22.61	69.66
**EOT**	0.05 to 1 m/yr ; **160 m**	13.81	38.64	23.79	76.24
**SHALLOW_MIO**	"	14.04	42.99	26.17	83.20
**DEEP_MIO**	"	12.85	35.61	22.34	70.80
**RBEDMAP2**	"	1.67	31.32	17.69	50.69
**EOT**	**PD - 1°C**; 160 m	13.82	37.97	23.91	75.70
**SHALLOW_MIO**	"	13.96	42.23	26.12	82.31
**DEEP_MIO**	"	12.76	35.38	22.45	70.59
**RBEDMAP2**	"	1.67	30.50	17.30	49.47
**EOT**	PD - 1°C; **120 m**	14.06	38.20	23.82	76.08
**SHALLOW_MIO**	"	14.02	42.65	26.49	83.15
**DEEP_MIO**	"	13.16	36.12	22.53	71.82
**RBEDMAP2**	"	14.29	33.61	23.93	71.83
**With basal sediments**					
**EOT**	0.05 to 1 m/yr ; 120 m	8.05	32.06	18.63	58.75
**SHALLOW_MIO**	"	8.71	32.80	19.25	60.77
**DEEP_MIO**	"	8.64	31.67	18.44	58.75
**RBEDMAP2**	"	11.74	32.32	20.53	64.59
**EOT**	0.05 to 1 m/yr ; **160 m**	8.15	32.02	18.65	58.82
**SHALLOW_MIO**	"	8.52	32.64	19.15	60.32
**DEEP_MIO**	"	8.39	31.09	18.37	57.85
**RBEDMAP2**	"	8.37	27.65	21.26	57.28
**EOT**	**PD - 1°C**; 160 m	8.48	31.40	19.10	58.97
**SHALLOW_MIO**	"	8.89	32.27	19.18	60.35
**DEEP_MIO**	"	8.73	30.39	18.42	57.53
**RBEDMAP2**	"	6.86	26.69	19.64	53.20
**EOT**	PD - 1°C; **120 m**	8.39	32.11	19.09	59.60
**SHALLOW_MIO**	"	9.36	33.36	19.78	62.49
**DEEP_MIO**	"	8.72	29.99	18.44	57.16
**RBEDMAP2**	"	12.17	30.35	20.67	63.19

**Full Late Pleistocene**	**Settings** b_{melt}; H_{coup}	**WAIS**	**EAIS_PACIFIC**	**EAIS_ATLANTIC**	**TOTAL**
**Standard simulations**					
**EOT**	0.05 to 1 m/yr ; 120 m	13.81	39.74	23.11	76.67
**SHALLOW_MIO**	"	14.35	40.49	22.57	77.41
**DEEP_MIO**	"	14.00	38.75	21.50	74.25
**RBEDMAP2**	"	15.67	37.09	23.83	76.59
**EOT**	0.05 to 1 m/yr ; **160 m**	14.21	39.44	22.94	76.59
**SHALLOW_MIO**	"	14.30	39.96	22.57	76.82
**DEEP_MIO**	"	9.75	35.74	19.88	65.36
**RBEDMAP2**	"	15.98	36.49	22.00	74.47
**EOT**	**PD - 1°C**; 160 m	13.46	38.18	22.58	74.22
**SHALLOW_MIO**	"	14.03	39.20	22.77	76.01
**DEEP_MIO**	"	12.72	32.82	21.49	67.03
**RBEDMAP2**	"	11.28	31.03	21.66	63.98
**EOT**	PD - 1°C; **120 m**	13.26	38.03	22.40	73.69
**SHALLOW_MIO**	"	13.52	38.29	22.65	74.47
**DEEP_MIO**	"	11.12	32.55	21.07	64.73
**RBEDMAP2**	"	4.68	29.34	17.74	51.56
**No GIA**					
**EOT**	0.05 to 1 m/yr ; 120 m	14.06	38.01	22.67	74.25
**SHALLOW_MIO**	"	14.08	36.59	21.80	72.46
**DEEP_MIO**	"	14.00	36.31	20.37	70.68
**RBEDMAP2**	"	5.98	32.87	17.44	56.29
**EOT**	0.05 to 1 m/yr ; **160 m**	13.67	37.01	22.15	72.83
**SHALLOW_MIO**	"	11.86	36.55	20.79	69.20
**DEEP_MIO**	"	9.92	33.47	18.03	61.41
**RBEDMAP2**	"	3.09	32.86	17.15	53.09
**EOT**	**PD - 1°C**; 160 m	13.44	36.54	22.01	72.00
**SHALLOW_MIO**	"	13.95	37.38	21.75	73.09
**DEEP_MIO**	"	14.01	34.57	20.89	69.46
**RBEDMAP2**	"	6.80	30.26	17.24	54.30
**EOT**	PD - 1°C; **120 m**	13.50	36.46	21.79	71.75
**SHALLOW_MIO**	"	13.63	37.16	21.91	72.70
**DEEP_MIO**	"	13.05	33.96	20.69	67.71
**RBEDMAP2**	"	4.61	29.90	17.23	51.74
**With basal sediments**					
**EOT**	0.05 to 1 m/yr ; 120 m	13.81	39.74	23.11	76.67
**SHALLOW_MIO**	"	11.49	36.59	20.61	68.69
**DEEP_MIO**	"	12.76	35.67	19.72	68.15
**RBEDMAP2**	"	15.20	35.27	21.55	72.02
**EOT**	0.05 to 1 m/yr ; **160 m**	14.21	39.44	22.94	76.59
**SHALLOW_MIO**	"	10.74	34.37	18.99	64.11
**DEEP_MIO**	"	10.01	33.58	18.12	61.72
**RBEDMAP2**	"	14.76	32.67	19.83	67.26
**EOT**	**PD - 1°C**; 160 m	13.46	38.18	22.58	74.22
**SHALLOW_MIO**	"	10.76	35.47	20.02	66.25
**DEEP_MIO**	"	10.78	30.98	19.22	60.98
**RBEDMAP2**	"	9.38	29.41	19.48	58.28
**EOT**	PD - 1°C; **120 m**	10.39	34.61	19.77	64.76
**SHALLOW_MIO**	"	10.71	34.81	20.10	65.61
**DEEP_MIO**	"	9.89	30.12	19.01	59.04
**RBEDMAP2**	"	5.61	28.51	16.80	50.93

Late Pleistocene (70 mbsl)	Settings b_{melt}; H_{coup}	WAIS	EAIS_PACIFIC	EAIS_ATLANTIC	TOTAL
**Standard simulations**					
**EOT**	0.05 to 1 m/yr ; 120 m	12.82	39.64	22.32	74.77
**SHALLOW_MIO**	"	13.38	40.19	22.10	75.67
**DEEP_MIO**	"	12.23	37.29	21.48	71.00
**RBEDMAP2**	"	10.11	33.63	20.30	64.04
**EOT**	0.05 to 1 m/yr ; **160 m**	13.14	39.21	22.29	74.65
**SHALLOW_MIO**	"	13.54	39.19	22.11	74.83
**DEEP_MIO**	"	7.43	34.70	19.50	61.63
**RBEDMAP2**	"	10.04	32.58	19.71	62.33
**EOT**	**PD - 1°C**; 160 m	13.10	38.06	22.29	73.45
**SHALLOW_MIO**	"	13.25	37.22	22.25	73.73
**DEEP_MIO**	"	10.95	31.88	20.89	63.73
**RBEDMAP2**	"	5.06	29.68	17.09	51.82
**EOT**	PD - 1°C; **120 m**	12.98	37.72	22.16	72.87
**SHALLOW_MIO**	"	12.73	36.95	22.18	71.87
**DEEP_MIO**	"	9.99	31.27	20.56	61.83
**RBEDMAP2**	"	2.79	28.97	17.21	48.98
**No GIA**					
**EOT**	0.05 to 1 m/yr ; 120 m	13.14	37.64	21.45	72.22
**SHALLOW_MIO**	"	13.56	38.47	21.84	73.86
**DEEP_MIO**	"	10.22	35.50	18.80	64.51
**RBEDMAP2**	"	3.99	30.90	16.58	51.48
**EOT**	0.05 to 1 m/yr ; **160 m**	13.41	36.86	21.66	71.93
**SHALLOW_MIO**	"	11.17	36.50	19.96	67.63
**DEEP_MIO**	"	7.87	33.18	17.82	58.87
**RBEDMAP2**	"	1.89	30.60	16.24	48.73
**EOT**	**PD - 1°C**; 160 m	13.14	36.37	21.48	70.99
**SHALLOW_MIO**	"	13.73	36.62	21.58	71.93
**DEEP_MIO**	"	13.16	33.16	20.48	66.79
**RBEDMAP2**	"	4.56	29.81	16.76	51.13
**EOT**	PD - 1°C; **120 m**	13.11	36.18	21.48	70.77
**SHALLOW_MIO**	"	13.40	36.38	21.60	71.39
**DEEP_MIO**	"	12.55	32.35	20.40	65.30
**RBEDMAP2**	"	3.61	29.46	17.38	50.46
**With basal sediments**					
**EOT**	0.05 to 1 m/yr ; 120 m	10.53	35.93	19.67	66.13
**SHALLOW_MIO**	"	11.04	35.63	19.69	66.36
**DEEP_MIO**	"	11.82	34.52	19.53	65.86
**RBEDMAP2**	"	9.90	32.10	18.58	60.58
**EOT**	0.05 to 1 m/yr ; **160 m**	10.79	34.68	19.65	65.12
**SHALLOW_MIO**	"	11.16	35.14	19.62	65.93
**DEEP_MIO**	"	8.87	31.13	17.81	58.81
**RBEDMAP2**	"	8.31	31.50	16.82	56.62
**EOT**	**PD - 1°C**; 160 m	10.30	34.38	19.60	64.29
**SHALLOW_MIO**	"	10.61	35.03	19.93	65.86
**DEEP_MIO**	"	9.56	29.94	18.96	58.47
**RBEDMAP2**	"	3.93	28.53	15.87	48.33
**EOT**	PD - 1°C; **120 m**	10.25	34.23	19.88	64.35
**SHALLOW_MIO**	"	10.12	33.93	19.91	63.96
**DEEP_MIO**	"	8.16	29.40	18.19	55.76
**RBEDMAP2**	"	2.55	27.69	16.01	46.25

References

Alvarez-Solas, J., et al., 2011: Heinrich event 1: an example of dynamical ice-sheet reaction to oceanic changes. *Clim. Past*, **7**, 1297–1306.

Arndt, J. E., et al., 2013: The International Bathymetric Chart of the Southern Ocean (IBCSO) Version 1.0?A new bathymetric compilation covering circum-Antarctic waters. *Geophys. Res. Lett.*, **40 (12)**, 3111–3117.

Barrett, P. and P. Froggatt, 1978: Densities, porosities, and seismic velocities of some rocks from Victoria Land, Antarctica. *New Zealand journal of geology and geophysics*, **21 (2)**, 175–187.

Bougamont, M., J. L. Bamber, J. K. Ridley, R. M. Gladstone, W. Greuell, E. Hanna, A. J. Payne, and I. Rutt, 2007: Impact of model physics on estimating the surface mass balance of the Greenland ice sheet. *Geophys. Res. Lett.*, **34 (17)**.

Brady, E. C., B. L. Otto-Bliesner, J. E. Kay, and N. Rosenbloom, 2013: Sensitivity to glacial forcing in the CCSM4. *J Climate*, **26 (6)**, 1901–1925.

Brancolini, G., A. K. Cooper, and F. Coren, 1995: Seismic facies and glacial history in the western Ross Sea (Antarctica). *Geology and Seismic Stratigraphy of the Antarctic Margin*, 209–233.

Busetti, M., G. Spadini, F. M. Van der Wateren, S. Cloetingh, and C. Zanolla, 1999: Kinematic modelling of the west Antarctic rift system, Ross Sea, Antarctica. *Global Planet. Change*, **23 (1-4)**, 79–103.

Cochrane, G. R., L. De Santis, and A. K. Cooper, 1995: Seismic velocity expression of glacial sedimentary rocks beneath the ross sea from sonobuoy seismic-refraction data. *Geology and Seismic Stratigraphy of the Antarctic Margin*, 261–270.

Cooper, A. K., P. F. Barker, G. Brancolini, and M. Hambrey, 1995: *Geology and seismic stratigraphy of the Antarctic margin*, Vol. 68. American Geophysical Union.

De Santis, L., J. B. Anderson, G. Brancolini, and I. Zayatz, 1995: Seismic record of late oligocene through miocene glaciation on the central and eastern continental shelf of the ross sea - geology and seismic stratigraphy of the antarctic margin. *Antarct. Res. Ser.*, 235–260.

De Santis, L., J. B. Anderson, G. Brancolini, and I. Zayatz, 1997: Glaciomarine deposits on the continental shelf of ross sea, antarctica. *Glaciated Continental Margins*, Springer, 110–113.

De Santis, L., S. Prato, G. Brancolini, M. Lovo, and L. Torelli, 1999: The Eastern Ross Sea continental shelf during the Cenozoic: implications for the West Antarctic ice sheet development. *Global Planet. Change*, **23 (1)**, 173–196.

DeConto, R. and D. Pollard, 2016: Contribution of Antarctica to past and future sea-level rise. *Nature*, **531 (7596)**, 591–597.

Dunbar, G., et al., 2007: Late Pleistocene to Holocene strata from soft-sediment coring at the AND-1B Site, ANDRILL McMurdo Ice Shelf Project, Antarctica. *Terra Antartica*, **14 (2)**, 141–154.

Dunbar, G. B., C. Atkins, D. Magens, and F. Niessen, 2009: Physical Properties of the AND-2A Core, ANDRILL Southern McMurdo Sound Project, Antarctica. *Terra Antartica, 15*, **1**, 49–56.

Fitzgerald, P. and S. Baldwin, 1997: Detachment fault model for the evolution of the ross embayment. *The Antarctic Region: Geological Evolution and Processes*, 555–564.

Gasson, E., R. M. DeConto, D. Pollard, and R. H. Levy, 2016: Dynamic Antarctic ice sheet during the early to mid-Miocene. *P. Natl. Acad. Sci. USA*, **113 (13)**, 3459–3464.

Golledge, N. R., D. E. Kowalewski, T. R. Naish, R. H. Levy, C. J. Fogwill, and E. Gasson, 2015: The multi-millennial Antarctic commitment to future sea-level rise. *Nature*, **526 (7573)**, 421–425.

Granot, R., S. Cande, J. Stock, F. Davey, and R. Clayton, 2010: Postspreading rifting in the Adare Basin, Antarctica: regional tectonic consequences. *Geochem. Geophy. Geosy.*, **11 (8)**.

Harwood, D., et al., 2008: Synthesis of the Initial Scientific Results of the ANDRILL Southern McMurdo Sound Project, Victoria Land Basin, Antarctica. *Studies from the ANDRILL, Southern McMurdo Sound Project, Antarctica. Terra Antart*, **15**.

Hayes, D. and L. Frakes, 1975: General synthesis, deep sea drilling project leg 28. *Initial Reports of the Deep Sea Drilling Project*, **28**, 919–942.

Henrys, S., C. Bücker, L. Bartek, S. Bannister, F. Niessen, and T. Wonik, 2000: Correlation of seismic reflectors with CRP2/2A, Victoria Land Basin, Antarctica. *Terra Antartica*, **7 (3)**, 221–230.

Hutter, K., 1983: *Theoretical glaciology: material science of ice and the mechanics of glaciers and ice sheets.* Reidel Publishing Company, Dordrecht, The Netherlands.

Kim, S., et al., 2018: Seismic stratigraphy of the Central Basin in northwestern Ross Sea slope and rise, Antarctica: Clues to the late Cenozoic ice-sheet dynamics and bottom-current activity. *Mar. Geol.*, **395**, 363–379.

Krinner, G. and C. Genthon, 1999: Altitude dependence of the ice sheet surface climates. *Geophys. Res. Lett.*, **26**, 2227–2230.

Kusznir, N., S. Stovba, R. Stephenson, and K. Poplavskii, 1996: The formation of the northwestern Dniepr-Donets Basin: 2-D forward and reverse syn-rift and post-rift modelling. *Tectonophysics*, **268 (1-4)**, 237–255.

Laske, G. and G. Masters, 1997: A Global Digital Map of Sediment Thickness. *EOS Trans. AGU*, **78**.

Lemeur, E. and P. Huybrechts, 1996: A comparison of different ways of dealing with isostasy: examples from modeling the Antarctic ice sheet during the last glacial cycle. *Ann. Glaciol.*, **23**, 309–317.

Lindeque, A., K. Gohl, F. Wobbe, and G. Uenzelmann-Neben, 2016: Preglacial to glacial sediment thickness grids for the Southern Pacific Margin of West Antarctica. *Geochem. Geophy. Geosy.*, **17 (10)**, 4276–4285.

Luyendyk, B., D. Wilson, and C. Siddoway, 2003: Eastern margin of the Ross Sea Rift in western Marie Byrd Land, Antarctica: Crustal structure and tectonic development. *Geochem. Geophy. Geosy.*, **4 (10)**.

MacAyeal, D., 1989: Large-Scale Ice Flow Over a Viscous Basal Sediment: Theory and Application to Ice Stream B, Antarctica. *J. Geophys. Res.*, **94**, 4071–4087.

Maule, C. F., M. E. Purucker, N. Olsen, and K. Mosegaard, 2005: Heat flux anomalies in Antarctica revealed by satellite magnetic data. *Science*, **309 (5733)**, 464–467.

McKay, R., L. De Santis, and D. Kulhanek, 2017: Expedition 374 Scientific Prospectus: Ross Sea West Antarctic Ice Sheet History. *Int. Ocean Discov. Program.*

Naish, T., et al., 2008: Late Cenozoic climate history of the Ross Embayment from the AND-1B drill hole: Culmination of three decades of Antarctic margin drilling. *Antarctica: A Keystone in a Changing World. Proceedings of the 10th International Symposium on Antarctic Earth Sciences*, 71–82.

Niessen, F., D. Magens, and A. Gebhardt, 2007: Physical properties of the AND-1B Core, ANDRILL McMurdo ice shelf project, Antarctica. *Terra Antartica*, **14 (3)**, 155–166.

Obase, T., A. Abe-Ouchi, K. Kusahara, H. Hasumi, and R. Ohgaito, 2017: Responses of Basal Melting of Antarctic Ice Shelves to the Climatic Forcing of the Last Glacial Maximum and CO2 Doubling. *J Climate*, **30 (10)**, 3473–3497.

Pattyn, F., et al., 2013: Grounding-line migration in plan-view marine ice-sheet models: results of the ice2sea MISMIP3d intercomparison. *J. Glaciol.*, **59 (215)**, 410–422.

Pekar, S., M. A. Speece, G. S. Wilson, D. S. Sunwall, and K. J. Tinto, 2013: The Offshore New Harbour Project: deciphering the Middle Miocene through Late Eocene seismic stratigraphy of Offshore New Harbour, western Ross Sea, Antarctica. *Geol. Soc. SP*, **381 (1)**, 199–213.

Petrini, M., et al., 2018: Interplay of grounding-line dynamics and sub-shelf melting during retreat of the Bjørnøyrenna Ice Stream. *Sci. Rep-UK*, **8 (1)**, 7196.

Peyaud, V., 2006: Role of the Ice Sheet Dynamics in major climate changes. Ph.D. thesis, Laboratoire de Glaciologie et de Géophysique de l'Environnement, Université Grenoble I, 420 pp pp.

Peyaud, V., C. Ritz, and G. Krinner, 2007: Modeling the early weichselian eurasian ice sheets: role of ice shelves and influence of ice-dammed lakes. *Clim. Past*, **3**, 375–386.

Pollard, D. and R. DeConto, 2012: Description of a hybrid ice sheet-shelf model, and application to Antarctica. *Geosci. Model Dev.*, **5 (5)**, 1273.

Pollard, D., R. M. DeConto, and R. B. Alley, 2015: Potential Antarctic Ice Sheet retreat driven by hydrofracturing and ice cliff failure. *Earth Planet Sc Lett*, **412**, 112–121.

Reeh, N., 1991: Parameterization of melt rate and surface temperature on the greenland lce sheet. *Polarforschung*, **5913**, 113–128.

Ritz, C., V. Rommalaere, and C. Dumas, 2001: Modeling the evolution of antarctic ice sheet over the last 420,000 years: Implications for altitude changes in the vostok region. *J. Geophys. Res.*, **106**, 31 943–31 964.

Roberts, A. M., N. J. Kusznir, G. Yielding, and P. Styles, 1998: 2D flexural backstripping of extensional basins; the need for a sideways glance. *Petroleum Geoscience*, **4 (4)**, 327–338.

Rommelaere, V. and C. Ritz, 1996: A thermomechanical model of ice-shelf flow. *Ann. Glaciol.*, **23**, 13–20.

Sauli, C., M. Busetti, L. De Santis, and N. Wardell, 2014: Late Neogene geomorphological and glacial reconstruction of the northern Victoria Land coast, western Ross Sea (Antarctica). *Mar. Geol.*, **355**, 297–309.

Shapiro, N. and M. Ritzwoller, 2004: Inferring surface heat flux distributions guided by a global seismic model: particular ap- plication to Antarctica. *Earth Planet. Sci. Lett.*, **223**, 213–214.

Siddoway, C. S., S. L. Baldwin, P. G. Fitzgerald, C. M. Fanning, and B. P. Luyendyk, 2004: Ross Sea mylonites and the timing of intracontinental extension within the West Antarctic rift system. *Geology*, **32 (1)**, 57–60.

Sorlien, C. C., B. Luyendyk, D. Wilson, R. Decesari, L. Bartek, and J. Diebold, 2007: Oligocene development of the West Antarctic Ice Sheet recorded in eastern Ross Sea strata. *Geology*, **35 (5)**, 467–470.

Stearns, L. A., B. E. Smith, and G. S. Hamilton, 2008: Increased flow speed on a large East Antarctic outlet glacier caused by subglacial floods. *Nature Geosci.*, **1 (12)**, 827–831.

Steckler, M. and A. Watts, 1978: Subsidence of the Atlantic-type continental margin off New York. *Earth Planet. Sci. Lett.*, **41 (1)**, 1–13.

Steinhauff, D. and P. Webb, 1987: Miocene foraminifera from DSDP site 272, Ross Sea. *Antarctic Journal of the United States;(USA)*, **22 (5)**.

Team, C. R. S., 1998: Initial report on CRP-1, Cape Roberts Project, Antarctica. *Terra Antartica*, **5 (1)**.

Team, C. R. S., 1999: Initial Report on CRP-2/2A, Cape Roberts Project, Antarctica. *Terra Antartica*, **6**, 173.

Team, C. R. S., 2000: Studies from the cape roberts project, ross sea, antarctica, initial reports on crp-3. *Terra Antartica*, **7 (1)**.

Tesauro, M., M. K. Kaban, and S. A. Cloetingh, 2012: Global strength and elastic thickness of the lithosphere. *Global Planet. Change*, **90**, 51–57.

Van de Berg, W. J., M. Van Den Broeke, J. Ettema, E. Van Meijgaard, and F. Kaspar, 2011: Significant contribution of insolation to Eemian melting of the Greenland ice sheet. *Nat. Geosci.*, **4 (10)**, 679.

Wilson, D. S., S. S. Jamieson, P. J. Barrett, G. Leitchenkov, K. Gohl, and R. D. Larter, 2012: Antarctic topography at the Eocene–Oligocene boundary. *Palaeogeography Palaeoecol.*, **335**, 24–34.

Wilson, D. S. and B. P. Luyendyk, 2009: West Antarctic paleotopography estimated at the Eocene-Oligocene climate transition. *Geophys. Res. Lett.*, **36 (16)**.

Wright, A. and M. J. Siegert, 2011: The identification and physiographical setting of Antarctic subglacial lakes: An update based on recent discoveries. *Geophys. Monogr. Ser*, **192**, 9–26.

Wright, A., et al., 2012: Evidence of a hydrological connection between the ice divide and ice sheet margin in the Aurora Subglacial Basin, East Antarctica. *J Geophys. Res-Earth*, **117 (F1)**.

Zwally, H. J., M. B. Giovinetto, M. A. Beckley, and J. L. Saba, 2012: Antarctic and Greenland drainage systems. *GSFC Cryospheric Sciences Laboratory.*
